# MicroRNA-221 protects myocardial contractility in myocardial ischemia/reperfusion injury through phospholamban

**DOI:** 10.1371/journal.pone.0316887

**Published:** 2025-01-30

**Authors:** Hongyu Li, Jimiao Qiu, Chang Liu, Guobing Yu, Danyu Wu, Yichun Chu, Kai Wang

**Affiliations:** 1 School of Nursing, NingBo College of Health Sciences, Ningbo, Zhejiang, China; 2 Department of Health Service, 906 Hospital of Joint Logistic Support Force of PLA, Ningbo, Zhejiang, China; 3 Department of Respiratory Medicine, The Second Affiliated Hospital of Harbin Medical University, Harbin, Heilongjiang, China; 4 Department of Pathology, 906 Hospital of Joint Logistic Support Force of PLA, Ningbo, Zhejiang, China; The Open University, UNITED KINGDOM OF GREAT BRITAIN AND NORTHERN IRELAND

## Abstract

**Objective:**

To investigate the effects and mechanisms of miRNA 221 on myocardial ischemia/reperfusion injury (MIRI) in mice through the regulation of phospholamban (PLB) expression.

**Methods:**

The MIRI mouse model was created and mice were divided into sham, MIRI, MIRI+ 221, and MIRI+ scr groups, with miRNA 221 overexpression induced in the myocardium of MIRI mice by targeted myocardial injection. Quantitative RT-PCR analysis was performed to observe the variation in miRNA 221, PLB, SERCA2, RYR2, NCX1, Cyt C and caspase 3 mRNA levels in myocardium, while Western blot assessed the levels of PLB, p-PLB (Ser16), p-PLB (Thr17), SERCA2, RYR2, NCX1, Cyt C and caspase 3 proteins. Changes in the structural integrity of the mouse heart were identified with HE and MASSON staining, while TUNEL staining was used to evaluate the TUNEL-positive cells of cardiomyocytes. Changes in myocardium calcium concentration were detected with reagent kits and the targeting interaction between miRNA 221 and PLB was evaluated using a luciferase reporter assay.

**Results:**

In the myocardium of MIRI mice, miRNA 221 level was significantly reduced, while the levels of PLB, p-PLB (Ser16), p-PLB (Thr17), and apoptosis-related genes caspase 3, and Cyt C were increased markedly, as well as calcium levels in myocardium. Following the overexpression of miRNA 221 in myocardium, there was a marked alleviation of myocardial injury and cardiomyocyte apoptosis and necrosis, significant enhancement of left ventricular systolic function, and marked decrease in the levels of PLB, p-PLB (Ser16), p-PLB (Thr17), caspase 3 and Cyt C, as well as a significant decrease in total calcium levels in myocardium.

**Conclusions:**

miRNA 221 can alleviate myocardial injury in mouse myocardial ischemia/reperfusion by suppressing the expression of PLB, thus reducing calcium overload in myocardium.

## Introduction

Myocardial infarction is a leading cause of morbidity and mortality worldwide. Although thrombolytic therapy and primary Percutaneous Coronary Intervention (PCI) have been the most effective strategies for restoring blood flow to the ischemic myocardium, such processes can also cause ischemia-reperfusion injury. Ischemia-reperfusion injury is a complex pathological phenomenon that occurs when tissues undergo a period of ischemia, characterized by an inadequate blood supply and subsequent oxygen deprivation, followed by the restoration of perfusion. This re-establishment of blood flow, while essential for tissue recovery, paradoxically triggers a cascade of detrimental biochemical and cellular responses. These responses can exacerbate the initial ischemic damage or transform previously reversible cellular injuries into irreversible damage, thereby compounding the overall tissue injury. The mechanisms underlying ischemia-reperfusion injury involve oxidative stress, inflammatory responses, mitochondrial dysfunction, and calcium overload, all of which contribute to the exacerbation of tissue damage upon reperfusion. Myocardial ischemia/reperfusion injury (MIRI), primarily caused by coronary artery disease-induced blood supply obstruction, is a common occurrence after reperfusion therapy due to the high oxygen needs of the heart. Primary manifestations of MIRI include arrhythmias, myocardial diastolic dysfunction, and structural changes which ultimately result in death [1]. The mechanisms of MIRI are poorly understood, as its pathophysiology is complex and involves multiple factors [2].

MicroRNAs (miRNAs) are small endogenous RNAs ranging from 19 to 25 nucleotides in length. These RNAs exhibit a high degree of sequence conservation across eukaryotic cells and can effectively degrade or inhibit the translation of target mRNA encoded protein by binding to the 3’ untranslated region (3’UTR) of target mRNA molecules, resulting in silenced specific gene functions [3]. miRNAs have been reported to play a crucial role in various cardiovascular pathological processes, such as cardiac development, neovascularization, arrhythmias, myocardial hypertrophy, and chronic heart failure [4, 5]. Although several studies have shown the role of miRNA 221 in the regulation of cardiac structure and function [6–10], its involvement in MIRI and its underlying mechanisms have not been explored.

Phospholamban (PLB) is predominantly expressed in the sarcoplasmic reticulum of smooth muscle and cardiac muscle cells. Its primary function is to modulate the physiological activity of sarcoendoplasmic reticulum calcium-ATPase 2 (SERCA2), a calcium pump located on the membrane of the sarcoplasmic reticulum, through phosphorylation and dephosphorylation. Consequently, PLB plays a crucial role in the regulating of cardiac myocyte contraction [11]. However, our current understanding of the biological functions of PLB in MIRI and the underlying mechanism remain unknown. Thus, in this study, we aimed to validate the therapeutic effect of miRNA 221 on MIRI and explore whether the effect was functioned through regulation of its downstream target-PLB. Our study provided a potential therapeutic target for MIRI.

## Materials and methods

### Ethics statement and animals

24 male SPF-grade C57BL / 6J mice were provided by The Slac Laboratory Animal Center (8 weeks old; Shanghai Slac Laboratory Animal Co., Ltd., Shanghai, China). Feed was supplied by XIETONG.ORGANISM (China). The feeding environment maintained a temperature range of 20~25°C, relative humidity range of 40~70%. Before the test, the mice were acclimatized in the animal room environment for one week. All experimental procedures were performed in accordance with the National Institute of Health Guide for the Care and Use of Laboratory Animals. The experimental plan was approved by the Animal Research Committee of 906 hospital of joint logistic support force of PLA (Approval file number 2021SL003).

Methods of sacrifice: at the end of the experimental period, the mice were humanely euthanized by cervical dislocation after anesthesia. Methods of anesthesia: mice were weighed and intraperitoneally injected with 3% pentobarbital sodium at a dose of 45 mg per kilogram of body weight. Efforts to alleviate suffering: throughout the study, animals were monitored closely for signs of distress, pain, or discomfort. Any animal exhibiting abnormal behavior was immediately assessed and provided with additional analgesia or supportive care as needed by an investigator who had previous experience in setting up the in-vivo model and had completed courses in animal sciences and laboratory safety. Additionally, a 12-hour light/dark cycle was maintained, and food and water were provided ad libitum.

### In vivo MIRI model and miRNA 221 agomir delivery

24 mice were randomly divided into four groups: sham-operated group (sham group), MIRI injury group (MIRI group), MIRI injury + miRNA 221 agomir group (MIRI+ 221 group), MIRI injury + negative control sequence group (MIRI+ scr group), with 6 mice in each group.

The MIRI+221 group and the MIRI+scr groups were subjected to myocardial injection of miRNA 221 agomir and negative control (RiboBio, China), respectively. Specifically, five spots were selected on the myocardial surface of the left ventricle and intramyocardial injection was performed on the myocardial surface using a microinjector with a single-spot injection volume of approximately 3 nmol and a total injection volume of 15 nmol. The chest was closed after injection and the ischemia-reperfusion injury model was induced 24 hours later.

Mice were weighed and intraperitoneally injected with 3% pentobarbital sodium at a dose of 45 mg per kilogram of body weight. Transoral tracheal intubation was performed and a small animal ventilator was connected to adjust the respiratory rate to around 90–100 breaths/min and the tidal volume to 0.4–0.8 ml for assisted breathing. A multichannel physiological signal acquisition system was connected and II-lead electrocardiography (ECG) was recorded. Open heart surgery was performed on the third and fourth intercostal spaces of the left chest, and the left anterior descending coronary artery was ligated with an 8–0 silk suture to induce myocardial ischemia. Sham-operated mice received the same surgery without ligation. Thirty minutes after ligation, the ligature was cut and the heart regained blood perfusion. Successful ligation was confirmed by immediate elevation of the ST segment and a color change of the heart from red to gray. Three days after successful reperfusion in MIRI model mice, echocardiography was used to measure left ventricular ejection fraction (LVEF), left ventricular shortening fraction (LVFS), diastolic left ventricular internal diameter (LVIDd), systolic left ventricular internal diameter (LVIDs), Left Ventricular End-Diastolic Volume (LEDV), Left Ventricular End-Systolic Volume (LESV) and heart rate (HR). The mice were then euthanized, and their heart tissues were harvested for subsequent molecular and pathological analysis.

### Histology

The heart was embedded in paraffin and sectioned. The sections were then dewaxed and rehydrated before being immersed in a hematoxylin staining solution at room temperature for 5 minutes. The sections were then washed with tap water for 1 minute before briefly immersed in a 1% hydrochloric acid alcohol solution. Subsequently, the sections were washed with tap water until the tissue regained its blue coloration and subsequently immersed in an eosin staining solution for 3–5 minutes. After being dehydrated in ethanol and rendered transparent in xylene, the sections were sealed with neutral gum. Nuclei were observed under a light microscope as blue or violet-blue, while the cell pulp appeared pink and erythrocytes appeared more vividly red.

### miRNA stem-loop qPCR and mRNA qRT-PCR

Total RNA was extracted from the samples using the Trizol centrifuge column method (GENEray Biotechnology) and reverse transcription was used to synthesize cDNA. The PCR cycles used were: pre-denaturation at 95°C for 10 minutes, denaturation at 95°C for 10 seconds, and annealing at 60°C for 30 seconds for 40 cycles. The mRNA levels of each group were determined using the ChamQ SYBR Color qPCR Master Mix kit (Vazyme, China). The miRNA primers were synthesized by RiboBio (China), and the other Primers were synthesized by Tsingke (China). The primer sequences are shown in Table 1.

**Table 1:** Primer sequences for RT-qPCR.

Target gene	Sequence	product size(bp)	NCBI gene sequence accession number
PLB	Forward: 5’-CCAGTGAGCTTTCCTGCGTA-3’	166	NC_000076.7
	Reverse: 5’-ATAGCCGAGCGAGTGAGGTA-3’		
NCX1	Forward: 5’-AGACGGCTTGACAGAGGTTG-3’	118	NC_000083.7
	Reverse: 5’-AACAAGAGAGCCACCAGAGC-3’		
RYR2	Forward: 5’-AGGTGGCAGATGGCTCTCTA-3’	168	NC_000079.7
	Reverse: 5’-TTGAGGATGTTCCACCAGGC-3’		
SERCA2	Forward: 5’-GAACCTTTGCCGCTCATTTT-3’	156	NC_000071.7
	Reverse: 5’-AGGCTGCACACACTCTTTACC-3’		
caspase 3	Forward: 5’-CTGACTGGAAAGCCGAAACTC-3’	189	NC_000074.7
	Reverse: 5’-CGACCCGTCCTTTGAATTTCT-3’		
Cyt C	Forward: 5’-CCAAATCTCCACGGTCTGTTC-3’	107	NC_000072.7
	Reverse: 5’-ATCAGGGTATCCTCTCCCCAG-3’		
β-Actin	Forward: 5’-AGAGGGAAATCGTGCGTGAC-3’	189	NC_000071.7
	Reverse: 5’-CCAAGAAGGAAGGCTGGAAA-3’		
U6	Forward: 5’-CTCGCTTCGGCAGCACA-3’	91	NC_000083.7
	Reverse: 5’-ACGCTTCACGAATTTGCG-3’		

### Western blots

RIPA lysate (Beyotime Biotechnology) was used to extract total protein from heart tissues. After ultrasonic homogenization, centrifugation was performed at 11,000 g for 20 minutes. The concentration of the extracted protein was measured by employing the BCA method (Beyotime Biotechnology). 20 μg of protein were obtained from each group and separated through a 4–20% SurePAGE Bis-Tris gel (GenScript Biotech Corporation). The separated protein was then transferred to a PVDF membrane, blocked with 5% milk (or 5% BSA for phosphorylated protein) for an hour at room temperature and incubated overnight at 4°C with the appropriate primary antibody. The milk and BSA were diluted in 1×TBST buffer (0.05%, Tris (MW 121.14) 1.22g, NaCl 2.92g, Tween20 0.5ml, ddH_2_O 1000 ml). The secondary antibodies were used prior to signal development using the ECL kit (Beyotime Biotechnology) and analyzed using a gel imaging system (ChemiDoc XRS, Bio-RAD). β-Actin was used as the internal protein reference, and the ratio of the gray value of the target band to the internal reference band was taken as the relative expression of the protein [12].

The protocol for stripping is as follows: 1. After completing the Western chemiluminescence detection, rinse the membrane in distilled water for 5 minutes. 2. Add an appropriate amount of neutral Western primary and secondary antibody stripping buffer (Beyotime Biotechnology). Rinse on a shaker for 10 minutes. 3. Discard the stripping buffer and rinse the membrane 3 times with 1×TBST. 4. Proceed with blocking and other subsequent Western blotting steps.

The antibodies are as follows: PLB (ab219626,1:2000), p-PLB (Ser16) (ab92697,1:1000), p-PLB (Thr17) (bs-7483R,1:1000), Na^+^/Ca^2+^ exchangers 1 (NCX1) (ab177952, 1:1000), Cytochrome C (Cyt C) (ab110325, 1:1000), caspase 3 (bsm-33284RM, 1:1000), sarcoplasmic/endoplasmic reticulum Ca^2+^-ATPase 2 (SERCA2) (bs-6693R, 1:1000), Ryanodine receptor 2 (RYR2) (bs-6305R, 1:1000), β-Actin (AF5003, 1:1000), Goat anti-Rabbit IgG (GAR0072, 1:5000), Goat anti-Mouse IgG (GAR007, 1:5000). The antibodies were diluted in 5% milk (or 5% BSA for phosphorylated protein) in 1×TBST.

### TUNEL staining

The heart was embedded in paraffin and sectioned before stained using the TUNEL apoptosis in situ assay kit (ROCHE, Switzerland), following the corresponding protocol. The TUNEL-positive cells were subsequently observed under a light microscope after the slices were sealed. The number of TUNEL-positive cells was recorded and counted in three randomly selected field of view under 200× magnification for each sample. The final results for each group were expressed as the mean ± standard deviation ($\bar{x}\pm \mathrm{SD}$).

### The 3’UTR luciferase reporter assay

According to bioinformatics analysis from http://www.targetscan.org, the predicted binding site of miRNA 221 was found to be within the 197–203 nucleotide region of the PLB mRNA 3’UTR. Subsequently, wild-type and mutant vectors of PLB mRNA 3’UTR pGL3 reporter gene were constructed. These vectors were then cotransfected with miRNA 221 mimics biosynthesized (Biotend, China) into 293T cells for 24 hours, using the Effectene transfection kit (Qiagen, Germany). Subsequently, cells were lysed using lysis solution and the relative luciferase expression of the samples was measured using a dual-luciferase reporter gene assay kit (Promega, Singapore).

### Quantification of total calcium levels

The calcium content was measured using the Calcium Assay Kit (Njjcbio, China). Briefly, myocardial tissue samples were first homogenized in normal saline. The homogenates were then centrifuged to obtain the supernatant and the concentration of the supernatant was measured by employing the BCA method. Following the instructions provided with the reagent kit, an aliquot of the supernatant was mixed with the reagents to initiate a colorimetric reaction. The absorbance was measured using a spectrophotometer at a wavelength of 610nm. Calcium concentration was determined by comparing the absorbance values to a standard curve generated from known calcium standards.

### Statistics

GraphPad Prism 9 statistical software was used to perform computational analysis and generate plots. All data are presented as mean ± standard deviation ($\bar{x}\pm \mathrm{SD}$). When comparing two independent groups, we employed a two-tailed unpaired Student’s t-test for statistical analysis. For comparisons involving more than two groups, we first assessed the equality of variances using the Brown-Forsythe test. For data with equal variances, we utilized one-way ANOVA followed by Tukey’s multiple comparisons test. For data with unequal variances, we adopted the Brown-Forsythe and Welch ANOVA tests followed by Dunnett’s T3 multiple comparisons test. P < 0.05 was considered statistically significant.

## Results

### miRNA 221 is down-regulated in myocardium

**Fig 1:**
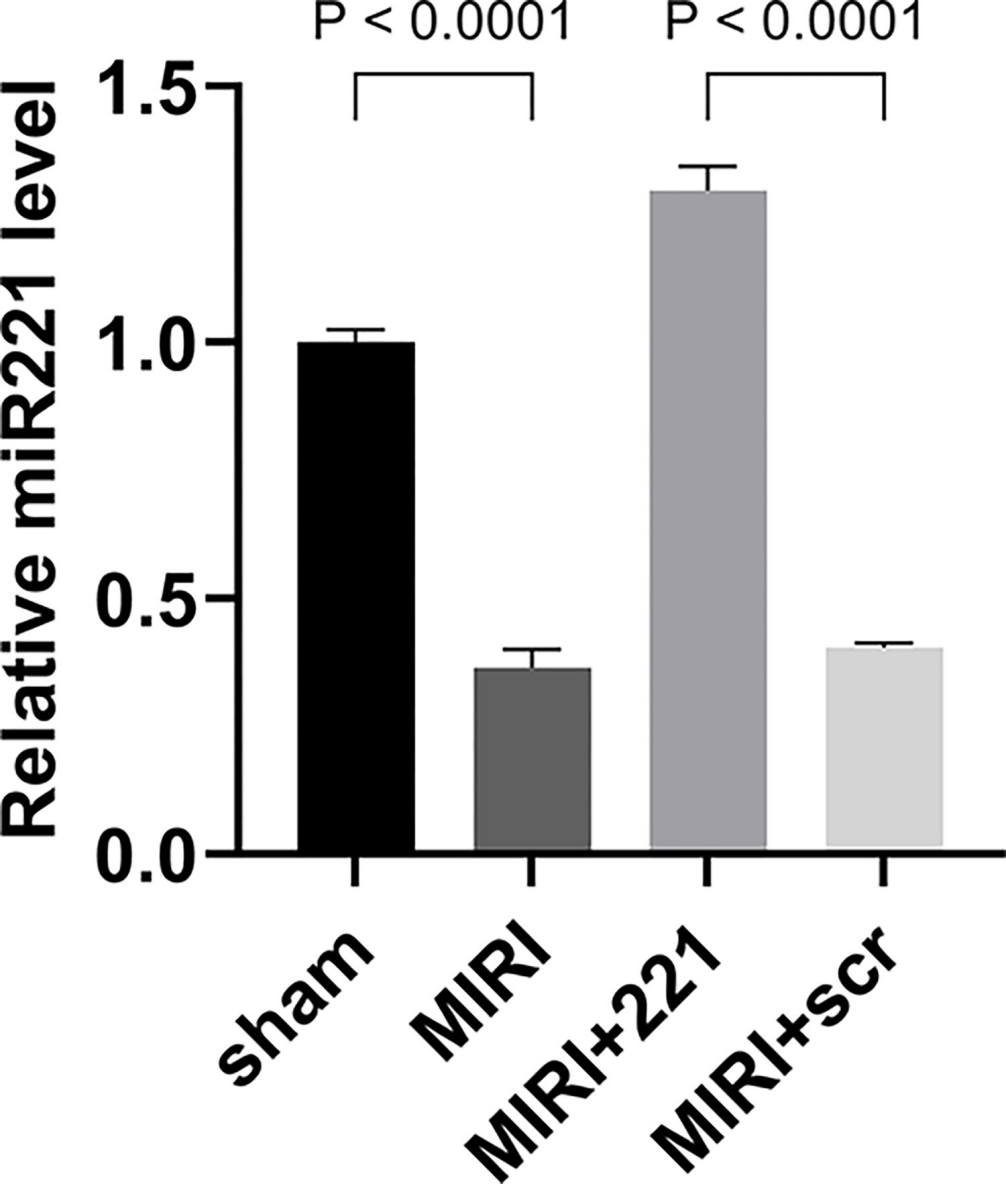
The level of miRNA 221 in the myocardium of MIRI mice was decreased. The myocardial tissues used for following detections were respectively treated with MIRI, MIRI + miRNA 221 agomir, MIRI+ negative control sequence. RT-PCR showed miRNA 221 levels were significantly diminished in the MIRI group. The results were presented by mean ± standard deviation and analyzed by one-way ANOVA followed by Tukey’s multiple comparisons test (n = 6).

To investigate the function of miRNA 221 in MIRI, the mouse model was established by ligating the left anterior descending coronary artery for thirty minutes and releasing for a 72h period for reperfusion. After MIRI the mice were euthanized to collect cardiac tissue for subsequent analysis. We found that miRNA 221 was significantly down-regulated in the MIRI group compared to the sham group (Fig 1), suggesting a marked decrease in miRNA 221 level in mouse hearts after MIRI. We then constructed a MIRI mouse model with miRNA 221 overexpression through myocardial injection. The results showed that the MIRI+221 group had a ~3-fold increase in miRNA 221 level compared to the MIRI group (Fig 1), indicating that we successfully established a miRNA 221 overexpressed MIRI mouse model.

### miRNA 221 alleviates myocardial injury and inhibits cardiomyocyte apoptosis and necrosis in MIRI mice

**Fig 2:**
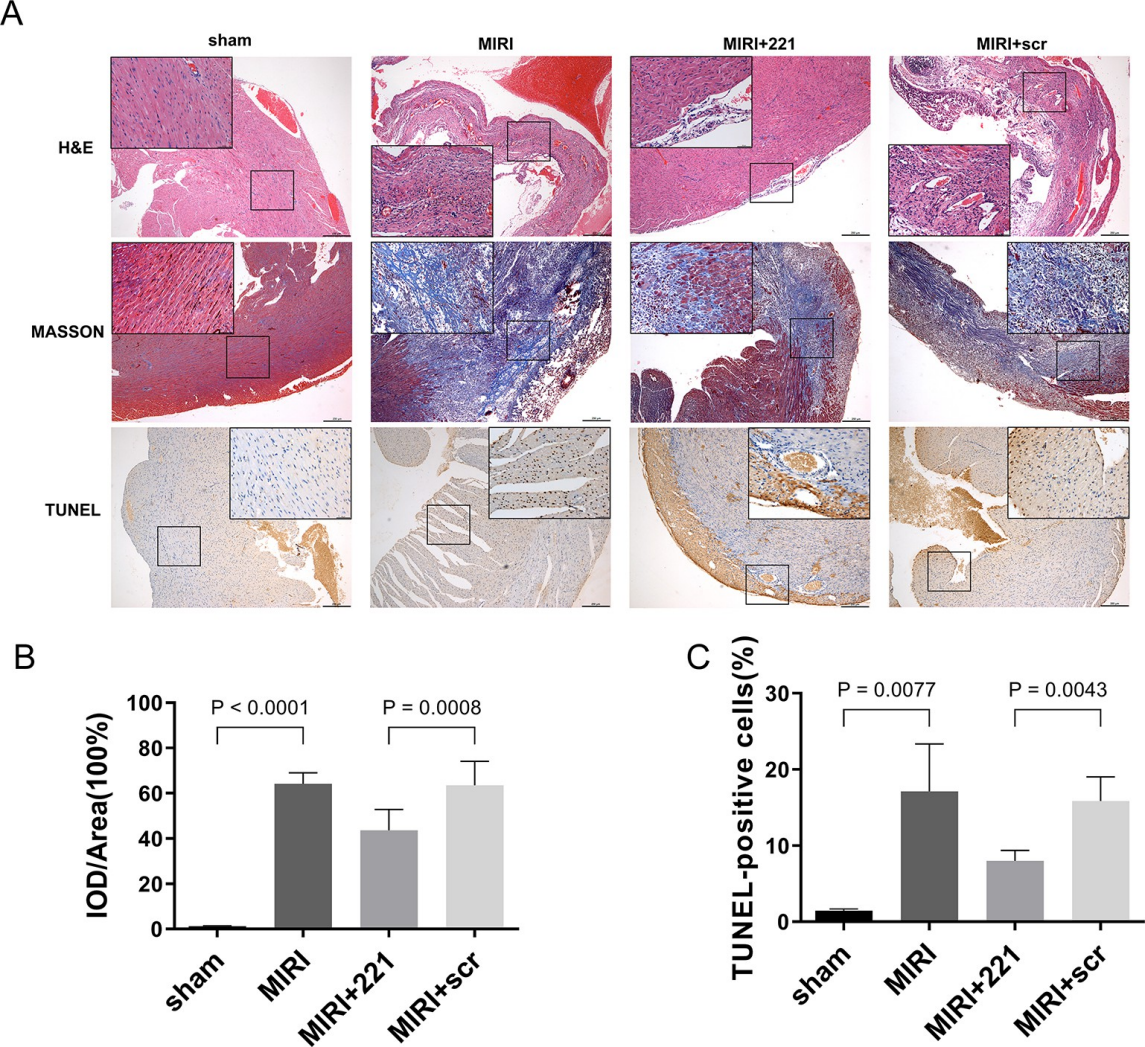
miRNA 221 alleviated myocardial injury and inhibited apoptosis and necrosis of cardiac cells in MIRI mice. (A) HE staining, Masson’s trichrome staining and TUNEL staining of mouse heart tissue sections. Scale bar: 250 μm. (inset: higher magnification; scale bar: 50 μm). (B) The myocardial fibrotic area of myocardial tissue of mice. The results were presented by mean ± standard deviation and analyzed by one-way ANOVA followed by Tukey’s multiple comparisons test (n = 6). (C) The TUNEL- positive cells of myocardial cells. The results were presented by mean ± standard deviation and analyzed by Brown-Forsythe and Welch ANOVA tests followed by Dunnett’s T3 multiple comparisons test. (n = 6).

To study the impact of miRNA 221 on the myocardial structure and function of MIRI mice, we used HE staining, Masson trichrome staining, and TUNEL staining to evaluate myocardial structure and apoptosis and necrosis of cardiomyocytes. HE staining showed that pathological changes in the heart in the MIRI group were more severe than in the Sham group. However, myocardial infarction in mice overexpressing miRNA 221 was significantly relieved (Fig 2A). This suggests that miRNA 221 overexpression in MIRI mice myocardium significantly alleviates myocardial injury. Subsequently, we used Masson trichrome staining to detect the degree of myocardial fibrosis and compare the differences in the fibrotic area. The results indicated that the heart of the MIRI group had a higher percentage of collagen fiber area compared to the sham group; conversely, the MIRI+221 group had a lower percentage compared to the MIRI+scr group (Fig 2A and 2B). This implies that after myocardial up-regulation of miRNA 221 in MIRI mice, the degree of fibrotic remodeling was markedly decreased. Finally, the results of the TUNEL staining showed that TUNEL-positive cells were significantly increased in the MIRI group compared to the sham group; TUNEL-positive cells were markedly reduced in the MIRI+221 group compared to the MIRI group (Fig 2A and 2C). This indicates that myocardial apoptosis and necrosis are significantly reduced after overexpression of miRNA 221 in myocardium of MIRI mice. In conclusion, we confirm that the overexpression of miRNA 221 in the myocardium of MIRI mice significantly alleviated both the extent of myocardial injury and cardiac cell death.

### miRNA 221 improves contractile function of the left ventricle in MIRI mice

**Fig 3:**
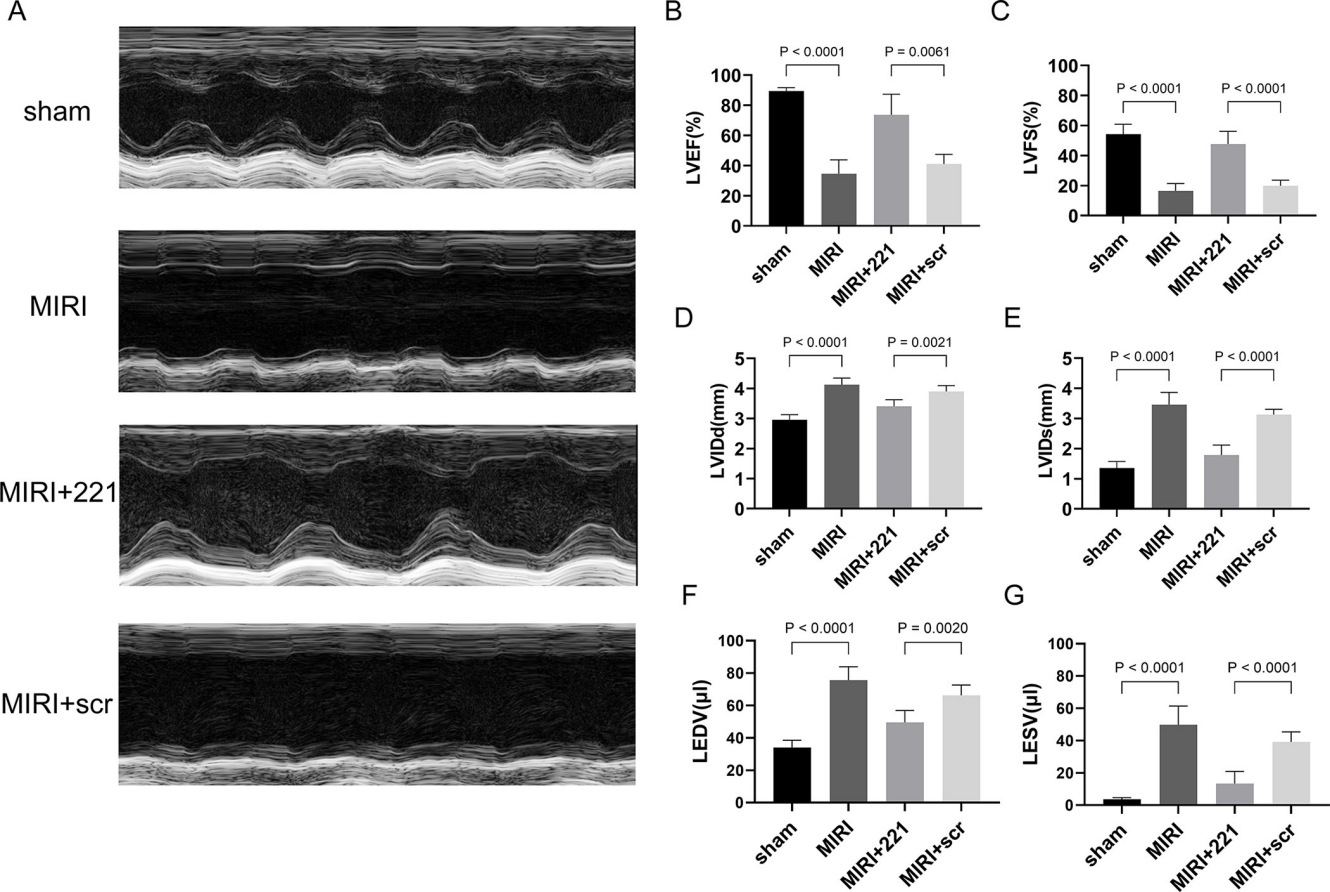
miRNA 221 improved left ventricular contractile function in MIRI mice. (A) Representative M-mode left ventricular echocardiographic images of mice. (B-E) In vivo assessment of heart function by echocardiography. The left ventricular ejection fraction (LVEF; B), left ventricular shortening fraction (LVFS; C), diastolic left ventricular internal diameter (LVIDd; D), systolic left ventricular internal diameter (LVIDs; E), Left Ventricular End-Diastolic Volume (LEDV; F), Left Ventricular End-Systolic Volume (LESV; G) of mice. The results were presented by mean ± standard deviation and analyzed by one-way ANOVA followed by Tukey’s multiple comparisons test (n = 6).

Three days after successful reperfusion in MIRI mice, color Doppler echocardiography was used to measure cardiac contractile function parameters. Compared to the control group, ischemia-reperfusion in mice resulted in a 61.4% and 69.6% decrease in LVEF and LVFS, with LVIDd and LVIDs increasing from 2.96±0.15mm and 1.35±0.22mm to 4.13±0.28mm and 3.45±0.41mm, LEDV and LESV increasing from 34.03±4.45μl and 3.64±1.02μl to 75.72±8.28μl and 49.90±11.5μl, respectively (Fig 3); After overexpression of miRNA 221 in myocardium, LVEF and LVFS in mice increased approximately 2–3 fold compared to the MIRI group, LVIDd and LVIDs decreased to 3.41±0.18mm and 1.79±0.32mm, and LEDV and LESV decreased to 49.54±7.43μl and 13.35±7.61μl, respectively (Fig 3). Heart rate was similar among the groups (S1 Fig). The results above suggest that left ventricular contractile function significantly improved in MIRI mice after myocardial overexpression of miRNA 221.

### miRNA 221 regulates cardiac function by directly inhibiting the expression of PLB in mouse myocardium of MIRI mice

**Fig 4:**
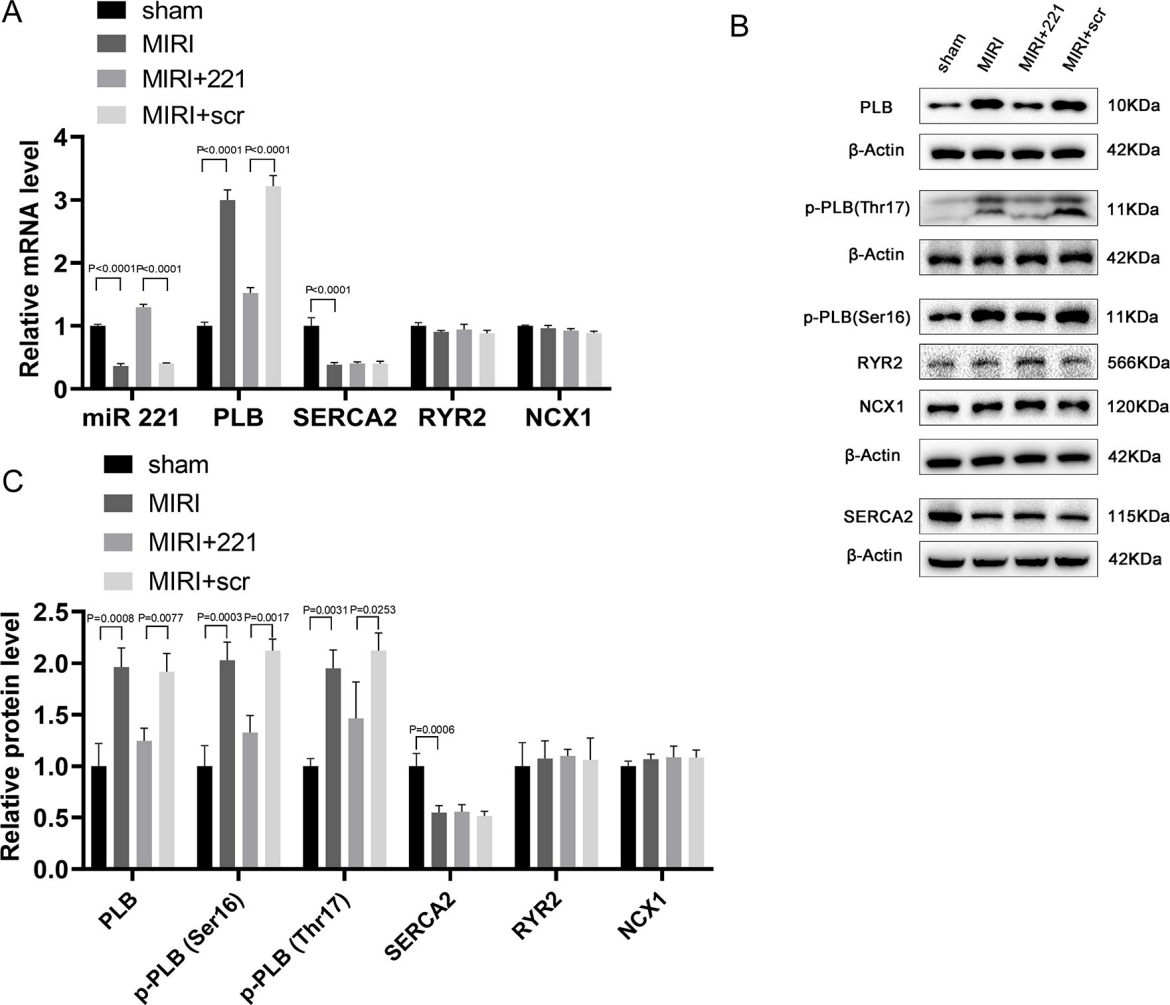
Altered expression of genes related to Ca^2+^ cycling or myocardial contraction. (A) mRNA expression of miR221, PLB, SERCA2, RYR2 and NCX1 in myocardial tissue of mice by RT-qPCR (n = 6). (B-C) Protein expression of PLB, p-PLB (Ser16), p-PLB (Thr17), SERCA2, RYR2 and NCX1 in the myocardial tissue of mice by western blot analysis (n = 3). The results were presented by mean ± standard deviation and analyzed by one-way ANOVA followed by Tukey’s multiple comparisons test.

To further explore the effects of miRNA 221 on the myocardium after MIRI, the expression of genes related to Ca^2+^ cycling or myocardial contraction were evaluated. Quantitative RT-PCR indicated that after MIRI, the mRNA level of SERCA2 was reduced by approximately 60% compared to the control group, while the PLB mRNA was ~3 fold that of the control group, with no significant changes in the RYR2 and NCX1 mRNA levels (Fig 4A). Western blot demonstrated that after MIRI, the protein level of SERCA2 decreased by approximately 50% compared to the control group, the PLB protein was approximately double the control, and there were no notable differences in RYR2 and NCX1 protein levels (Fig 4B and 4C). The regulation of SERCA2 activity during myocardial contraction is mainly through modulation of PLB phosphorylation and dephosphorylation. Further research revealed that phosphorylated PLB at Ser16 (the PKA site) or Thr17 (the CaMKII site) after MIRI was significantly increased in comparison to the control group (Fig 4B and 4C). After overexpressing miRNA 221 in myocardium, observable decreases in PLB, p-PLB (Ser16) and p-PLB (Thr17) levels were found compared with the MIRI group, with no marked changes in SERCA2, RYR2, and NCX1 levels (Fig 4). The above results suggest that miRNA 221 might regulate cardiac function by reducing the expression and activation of PLB in mouse myocardium.

**Fig 5:**
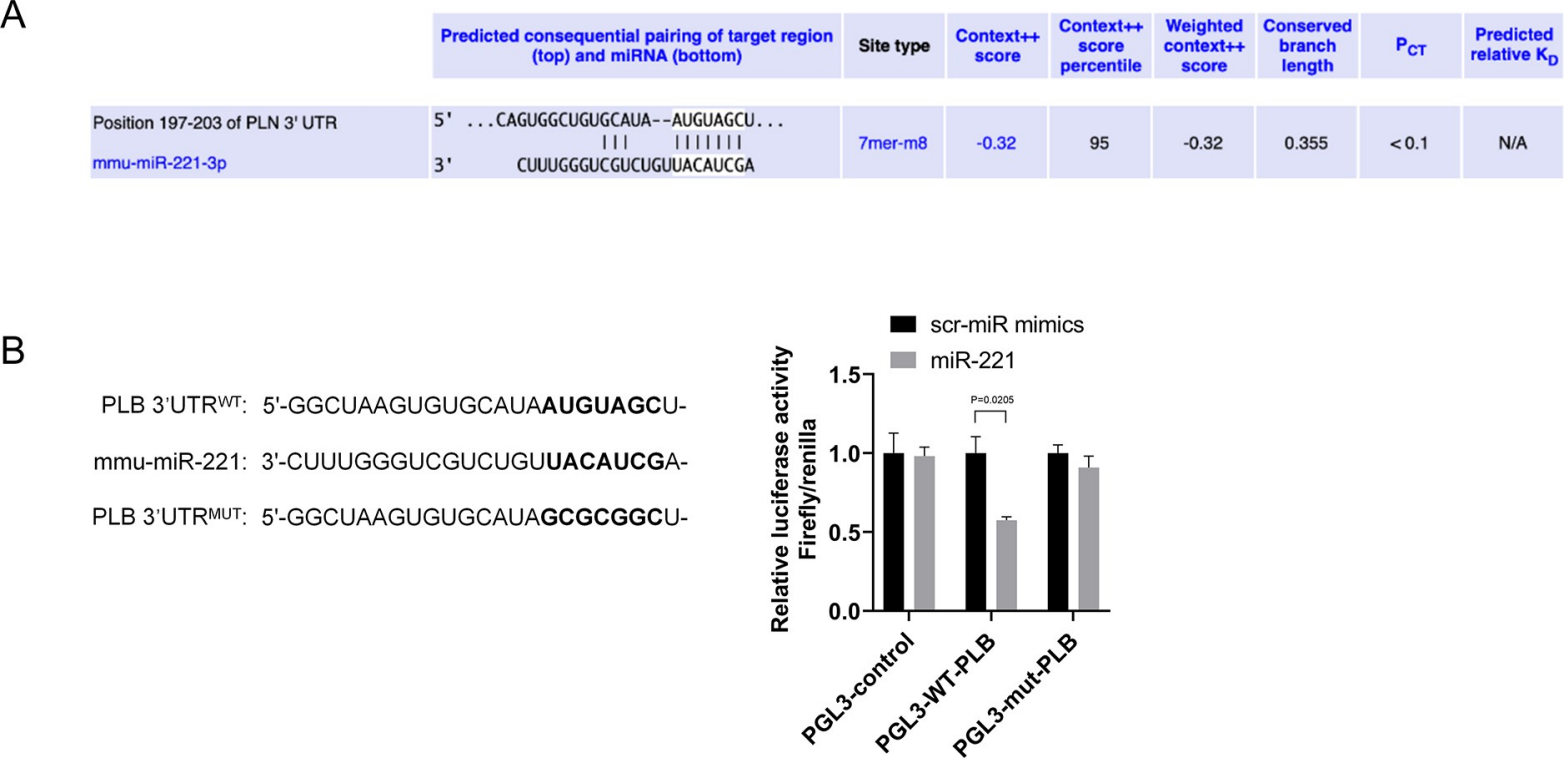
PLB is a target gene of miRNA 221. (A) The predicted binding sites of miRNA 221 on the 3′UTR sequence of PLB gene. (B) The luciferase activity of PGL3-control, PGL3-WT-PLB and PGL3-mut-PLB. Co-transfection of miRNA 221 mimics in HEK293 cells inhibited the activity of a luciferase reporter containing 478bp 3′UTR of mouse PLB gene. Mutations in the predicted miRNA 221 binding sites in the PLB 3’UTR eliminated the inhibitory effects of miRNA 221 mimics. The results of luciferase activity were presented by mean ± standard deviation and analyzed using the two-tailed unpaired Student’s t-test (n = 5).

Subsequently, through online analysis software, we discovered a specific binding region between the 3’UTR of the PLB mRNA and miRNA 221 sequence (Fig 5A), indicating that PLB is a potential target gene for miRNA 221. We then constructed a luciferase reporter containing 478bp of the 3’UTR of PLB mRNA with a predicted miRNA 221 binding site. Subsequent research using a dual luciferase reporter assay system revealed that compared to the control group, miRNA 221 significantly inhibited the luciferase activity of PLB 3’UTR, but had no significant effect on the luciferase activity of the PLB 3’UTR mut (Fig 5B). This result proves that miRNA 221 can specifically bind to PLB 3’UTR, thus downregulating the expression of the PLB gene at the post-transcriptional level.

### miRNA 221 alleviates calcium overload in myocardium and inhibits the expression of apoptosis-related genes in MIRI mice

**Fig 6:**
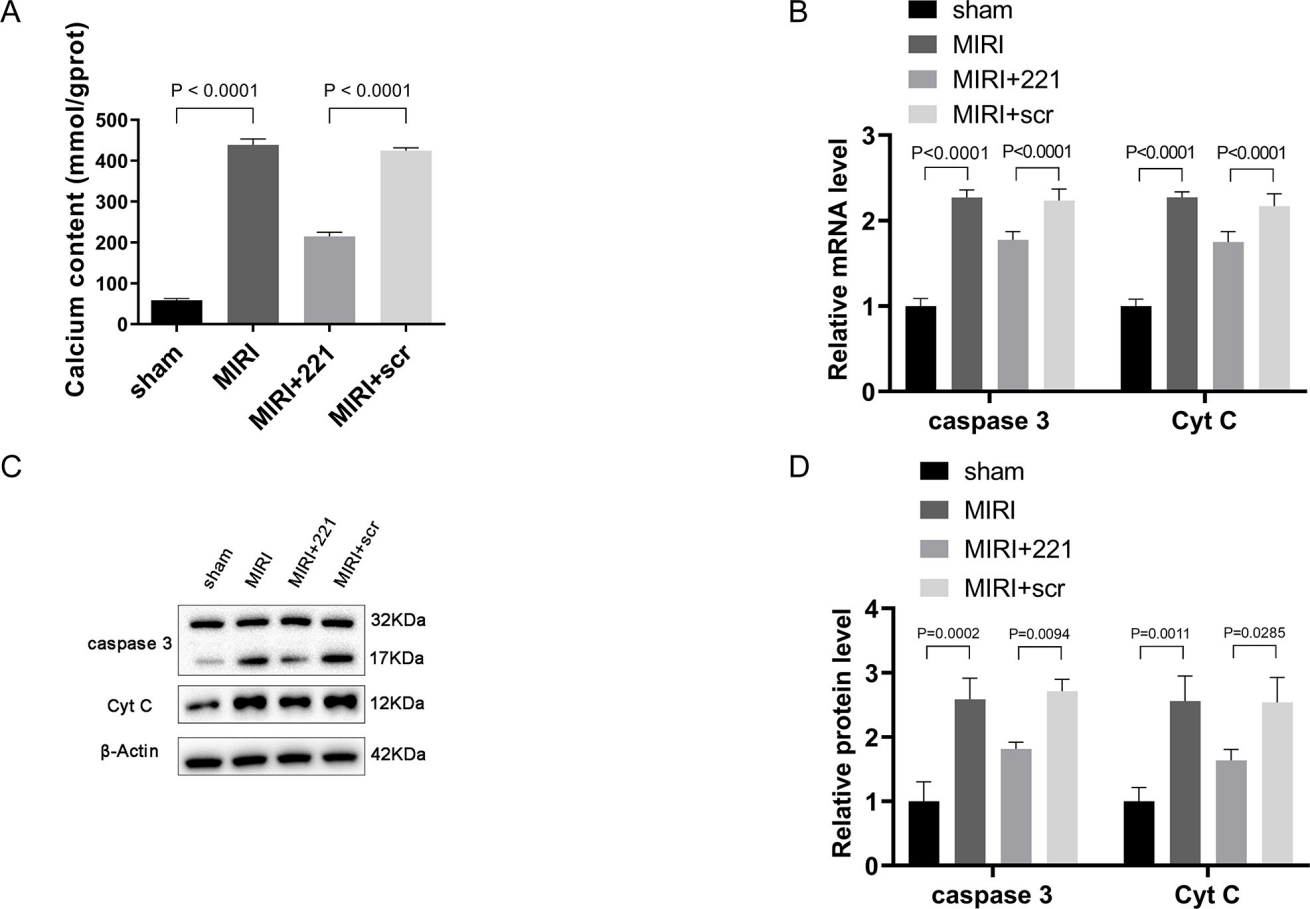
miRNA 221 alleviated calcium overload in myocardium and inhibited the expression of apoptosis-related genes in MIRI mice. (A) The calcium content in the myocardium of mice (n = 6). The results were presented by mean ± standard deviation and analyzed by the Brown-Forsythe and Welch ANOVA tests followed by Dunnett’s T3 multiple comparisons test. (B) mRNA expression of caspase3 and Cyt C in myocardial tissue of mice by RT-qPCR (n = 6). (C-D) Protein expression of caspase3 and Cyt C in the myocardial tissue of mice by western blot analysis (n = 3). The results were presented by mean ± standard deviation and analyzed by one-way ANOVA followed by Tukey’s multiple comparisons test.

Since PLB plays a crucial role in the regulation of intracellular Ca^2+^ concentration and Ca^2+^ levels in the cytoplasm of cardiomyocytes are significantly increased after MIRI, leading to calcium overload and myocardial damage, we first assessed the effect of miRNA 221 on calcium content in the myocardium of MIRI mice. The results showed that, compared to the Sham group, there was a marked increase in calcium content in the myocardium of the MIRI group, while overexpression of miRNA 221 led to a significant reduction in calcium content (Fig 6A). This result suggests that miRNA 221 can alleviate myocardium calcium overload by inhibiting the expression of PLB.

Calcium overload may trigger Cyt C release and the activation of apoptotic enzymes, precipitating the activation of the caspase cascade and inducing apoptosis. Therefore, we evaluated the expression of a group of genes closely related to apoptosis. The data indicated that, compared to the sham group, the mRNA levels of caspase 3 and Cyt C in the MIRI group increased approximately 2 to 2.5-fold; after overexpression of miRNA 221 in the myocardium, these levels decreased by approximately 20% compared to the MIRI group (Fig 6B). Western blot analysis showed that, compared to the sham group, protein levels of caspase 3 and Cyt C in the MIRI group increased approximately 2 to 2.5-fold; following myocardial overexpression of miRNA 221, there was a reduction of approximately 40% in these protein levels compared with the MIRI group (Fig 6C and 6D). These results suggest that miRNA 221 can relieve calcium overload and suppress the expression of apoptosis-related genes in myocardium of MIRI mice, thus protecting the myocardium.

## Discussion

MIRI injury refers to the process in which restoration of blood flow after cardiac vessel obstruction and subsequent myocardial ischemia may cause cardiomyocyte death and tissue damage. MIRI injury poses a severe problem for cardiac patients, as it exacerbates myocardial damage and worsens the condition during a cardiac event and the treatment period [1]. miRNA is a small noncoding RNA molecule of approximately 20–22 nucleotides that can mediate mRNA degradation or translation inhibition by binding to the mRNA of target genes, thus regulating gene expression. It plays an important regulatory role in many biological processes, such as cell proliferation, differentiation, apoptosis, and immune responses [3]. Due to the vital role of miRNA, it has become a focus of research in recent years. Some recent studies have found that miRNAs play a crucial role in the regulation of MIRI. Some miRNAs have also been upregulated in myocardium during MIRI, such as miRNA-1, miRNA-494, miRNA-21, miR-144-3p, etc. [10, 13–15]; While others are down-regulated, such as miRNA-133, miRNA-320 [13, 16].

The miRNA 221 gene sequence is located in Xp11.3 and is fully homologous in the core seed sequence between humans and mice. Early studies identified an increased level of miRNA 221 in the hearts of mice with cardiac hypertrophy models, which can promote the progression of cardiac hypertrophy by regulating the expression of p27 [9]; Zeki Ongen’s study in 27 patients diagnosed with acute coronary syndrome revealed a notable increase in the level of miRNA-221-3p in serum during the early phases, indicating that miRNA-221-3p can serve as an early predictive marker for acute coronary syndrome [6]. However, Robin Verjans et al. discovered that a lower level of miRNA 221 in the heart is associated with elevated myocardial fibrosis and heart failure in patients [8]; Xiaojun Liu et al. proved that miRNA 221 can inhibit pathological cardiac remodeling [7], and a recent study by Zhou et al. found that miRNA 221 can enhance myocardium viability and improve cardiac function post-myocardial infarction [10]; these studies suggested that miRNA 221 plays a protective role in cardiac function. Nevertheless, the role and underlying mechanisms of miRNA 221 in MIRI have not yet been explored. Our research finds that miRNA 221 level is significantly reduced in the cardiac tissue of MIRI mice, therefore, we hypothesize that miRNA 221 participates in MIRI.

To confirm this hypothesis, we successfully created a mouse model with myocardium overexpressing miRNA 221 by intramyocardial injection. The study found that miRNA 221 overexpression in mice significantly mitigated myocardial infarction compared to MIRI mice and markedly reduced myocardial fibrosis and cell death in MIRI mice. Subsequently, we further examined mouse cardiac function using color Doppler echocardiography and found that left ventricular systolic function of MIRI mice was significantly improved after miRNA 221 overexpression in the myocardium. This result suggested that miRNA 221 exerts a protective effect on MIRI. Therefore, our data support the idea that miRNA 221 plays a protective role in cardiac function.

Changes in cytoplasmic Ca^2+^ concentration play a critical role in the contraction and relaxation of cardiomyocytes. The sarcoplasmic reticulum releases a large amount of Ca^2+^ into the cytosol after activation of the ryanodine receptor, leading to cardiomyocyte contraction, while the SERCA2 retrieves Ca^2+^ from the cytoplasm into the sarcoplasmic reticulum, which induces cardiomyocyte relaxation [11, 17]. PLB is a calcium regulatory protein that directly modulates SERCA2 and plays a vital role in the regulation of intracellular Ca^2+^ concentration. PLB possesses two phosphorylation sites: Serine-16, which is phosphorylated by cAMP-dependent protein kinase, and Threonine-17, which is phosphorylated by Ca^2+^/calmodulin-dependent protein kinase II. Notably, phosphorylation of Serine-16 serves as a prerequisite for the phosphorylation of Threonine-17, as impairment in Serine-16 phosphorylation results in the ineffective phosphorylation of Threonine-17. In its non-phosphorylated state, PLB inhibits the function of SERCA2. However, upon phosphorylation by cAMP-dependent protein kinase A and/or Ca^2+^/calmodulin-dependent protein kinase II, this inhibitory effect of PLB on SERCA2 is abolished [11, 18]. In transgenic mice that overexpressing PLB, myocardial contractility and cardiac pump function were gradually impaired, ultimately leading to heart failure [19]. In contrast, PLB-knockout mice showed better cardiac function in the heart failure model compared to normal mice [20]. It has also been indicated that structural and functional changes of PLB could directly or indirectly affect the onset and progression of cardiovascular diseases such as dilated cardiomyopathies and heart failure, making it a potential therapeutic target for these diseases [21, 22].

However, the role of PLB in MIRI has not yet been clearly defined. Here, we found that in the cardiac tissue of MIRI mice, levels of PLB, p-PLB (Ser16) and p-PLB (Thr17) were significantly elevated, and both total PLB and p-PLB expressions increased to a similar extent compared to the control group. However, after miRNA 221 overexpression, levels of PLB, p-PLB (Ser16) and p-PLB (Thr17) were significantly reduced, while the reduction in both total PLB and p-PLB expressions was also nearly identical compared to the MIRI group. Therefore, we posit that no changes in PLB phosphorylation occur among the different groups and the alterations in p-PLB expression in myocardial tissue of MIRI are primarily attributed to changes in the level of total PLB due to the changes of miRNA 221 expression. Other signaling factors involved in intracellular Ca^2+^ cycling and myocardial contraction, such as RYR2 and NCX1, showed no significant differences after myocardial ischemia-reperfusion. SERCA2 was significantly reduced after MIRI, consistent with previous studies [23], while it showed no significant deviation from the control group after overexpression of miRNA 221. Subsequent in vitro experiments we confirmed that miRNA 221 could directly bind to the 3’UTR of PLB mRNA, exerting a targeted effect on PLB. Our results indicated that miRNA 221 primarily improves cardiac function in MIRI by regulating PLB.

MIRI is a complex pathophysiological process that involves multiple factors. The mechanisms of MIRI are far from clear, and calcium overload plays a crucial role [24]. Calcium overload refers to an increase in intracellular Ca^2+^ content and abnormalities in cellular Ca^2+^ transport mechanisms caused by various reasons, leading to structural damage to the cell and functional metabolic disorders. Currently, prevention and treatment of calcium overload, such as the use of calcium channel blockers, have become important therapeutic approaches for clinical intervention in MIRI [25]. The mechanisms by which calcium overload causes bodily injury have yet to be fully elucidated and mainly include: ①energy metabolism disorder; ②degradation of cell membranes and structural proteins; ③exacerbation of acidosis [26]. Studies have shown that calcium overload can trigger the opening of the mitochondrial permeability transition pore (mPTP), resulting in the release of Cyt C and activation of apoptotic proteases, activating the caspase cascade response and inducing cell apoptosis. The opening of mPTP is one of the important causes of MIRI [27–29]. Our study found that in MIRI mouse myocardium, the calcium content and mRNA and protein levels of caspase 3 and Cyt C were significantly increased. However, after overexpression of miRNA 221, the calcium content and mRNA and protein levels of caspase 3 and Cyt C were significantly decreased. This date indicates that in MIRI miRNA 221 can alleviate calcium overload, repress the expression of genes related to apoptosis, mitigate cell apoptosis and necrosis, and thereby protect the heart.

Research has established that the SERCA2/PLB complex serves as a pivotal regulator of myocardial contraction, responsible for transporting approximately 70% of Ca^2+^ in the human heart. Mutations in SERCA2 and PLB, as well as abnormal Ca^2+^ transport, can lead to contractile dysfunction and various cardiac pathologies [30]. Consequently, the SERCA2/PLB ratio directly reflects the strength of the calcium reuptake capacity in cardiomyocytes. A higher ratio indicates relatively enhanced SERCA2 activity, enabling cardiomyocytes to more efficiently pump cytosolic Ca^2+^ back into the sarcoplasmic reticulum, thereby maintaining intracellular Ca^2+^ homeostasis and protecting cardiomyocytes from calcium overload and associated damages. Conversely, a lower ratio signifies stronger inhibition of SERCA2 activity by PLB, resulting in diminished calcium reuptake capacity of cardiomyocytes, which predisposes them to calcium overload and myocardial injury. In our study, the SERCA2/PLB ratio underwent a marked reduction following MIRI, underscoring the disruption of calcium homeostasis in this pathological condition. Conversely, the overexpression of miRNA 221 in MIRI mice myocardium led to a significant increase in the SERCA2/PLB ratio, indicating a regulatory mechanism through which miRNA 221 may contribute to restoring calcium regulation and potentially mitigating the deleterious effects of MIRI. This observation highlights the promising therapeutic avenues for miRNA 221 modulation in addressing the sequelae of MIRI, with the potential to promote cardio protection and functional recovery. However, our research has generated conflicting findings regarding the observed elevation in the SERCA2/PLB ratio which facilitates enhanced calcium reuptake into sarcoplasmic reticulum, coupled with the decreased total intracellular calcium content in the MIRI+221 group. We hypothesize that the observed phenomenon can be attributed to the enhanced sarcoplasmic reticulum calcium reuptake capacity, which effectively alleviates calcium overload and myocardial cell damage. Consequently, this leads to a reduction in calcium influx, ultimately resulting in a decrease in the total intracellular calcium content in the MIRI+miR-221 group. However, the specific mechanisms underlying this phenomenon remain unclear. These inconsistences can be attributed to several factors, including but not limited to lesser mitochondrial Ca^2+^ uptake, lowered Ca^2+^ entry during each beat via L-type Ca^2+^ channels and increased Ca^2+^ efflux via NCX1 on account of potential differences in intracellular sodium concentration or NCX1 phosphorylation in the MIRI+221 group. Therefore, further investigation is warranted to measure the L-type Ca^2+^ current and Na^+^ /Ca^2+^ exchange current of isolated ventricular myocytes from mice among the different groups using the whole-cell patch-clamp technique.

The restoration or upregulation of miRNA 221 levels after MIRI may serve as a novel therapeutic strategy to mitigate the deleterious effects of PLB upregulation and resultant calcium overload. By targeting miRNA 221, we hypothesize that it could directly downregulate PLB expression, thereby normalizing cellular calcium homeostasis and reducing myocardial cell apoptosis and necrosis. Such an approach would require the development of safe and effective delivery systems for miRNA 221 mimics or enhancers, ensuring their stability and bioavailability in the ischemic myocardium. While miRNA 221 administration represents a direct approach to regulate PLB levels, an alternative strategy would be to target PLB itself through gene silencing techniques, such as using antisense oligonucleotides (ASOs) or CRISPR/Cas9-mediated gene editing. By specifically inhibiting PLB expression, we could potentially achieve similar therapeutic benefits as seen with miRNA 221 upregulation. However, the safety and efficacy of such therapies, particularly in the context of acute cardiac injury, must be rigorously evaluated in preclinical studies. Future studies should focus on validating the therapeutic potential of miRNA 221 modulation and PLB-directed gene silencing in preclinical models of MIRI. These investigations should incorporate detailed assessments of cardiac function, histological analysis of myocardial tissue, and measurements of calcium homeostasis to comprehensively evaluate the therapeutic effects. Moreover, the development of novel delivery systems tailored for cardiac tissue and the optimization of dosing strategies will be crucial for translating these findings into clinical applications. In conclusion, the potential therapeutic implications of our findings underscore the need for further research into the role of miRNA 221 and PLB in MIRI. By exploring both miRNA-based and gene-silencing strategies, we aim to uncover novel therapeutic avenues that could ultimately improve outcomes for patients suffering from acute myocardial infarction and its associated complications.

In conclusion, following MIRI injury, a reduction of miRNA 221 in myocardium leads to an up-regulation of the PLB level, which subsequently causes an elevation in cellular calcium levels, triggering calcium overload and ultimately resulting in increased myocardial cell apoptosis and necrosis and injury. This research has identified, for the first time, that miRNA 221 protects myocardial contractility in MIRI through PLB and has further elucidated a new mechanism of calcium overload in MIRI. These results could offer new therapeutic targets and strategies for the treatment of MIRI.

## Supporting information

S1 Fig The heart rate was similar among the groups. (TIF)

S1 Raw images (PDF)

S1 File (XLSX)
