# Supplementary figures and images for: MicroRNA-221 protects myocardial contractility in myocardial ischemia/reperfusion injury through phospholamban
Source: PLoS One. 2025 Jan 30;20(1):e0316887. doi: 10.1371/journal.pone.0316887 (PMC11781681; doi:10.1371/journal.pone.0316887)

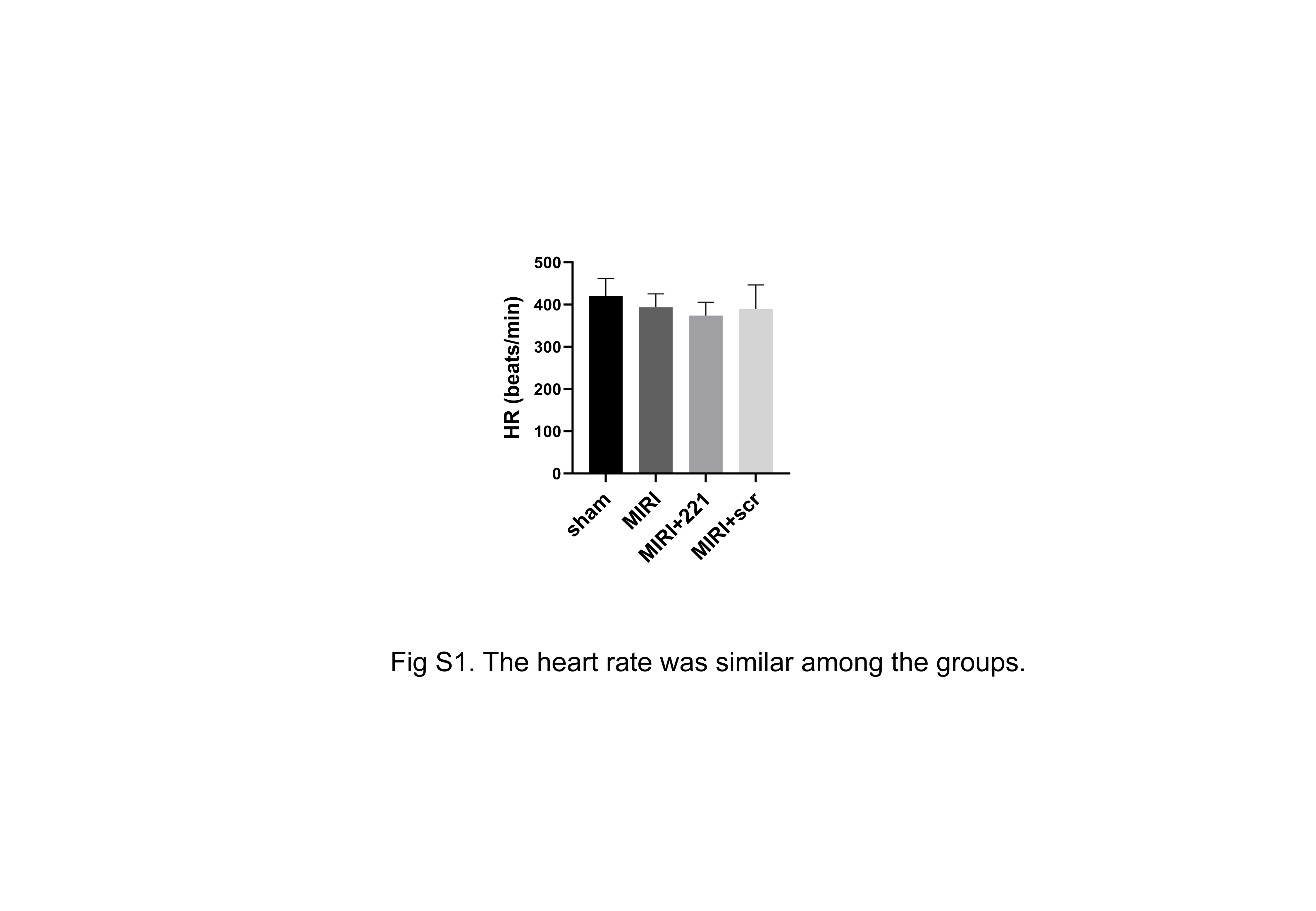

Supplement: S1 Fig — (TIF) [file pone.0316887.s001.tif]
