# Supplementary material for: MicroRNA-221 protects myocardial contractility in myocardial ischemia/reperfusion injury through phospholamban
Source: PLoS One. 2025 Jan 30;20(1):e0316887. doi: 10.1371/journal.pone.0316887 (PMC11781681; doi:10.1371/journal.pone.0316887)
Supplement: S1 Raw images — (PDF) [file pone.0316887.s002.pdf]

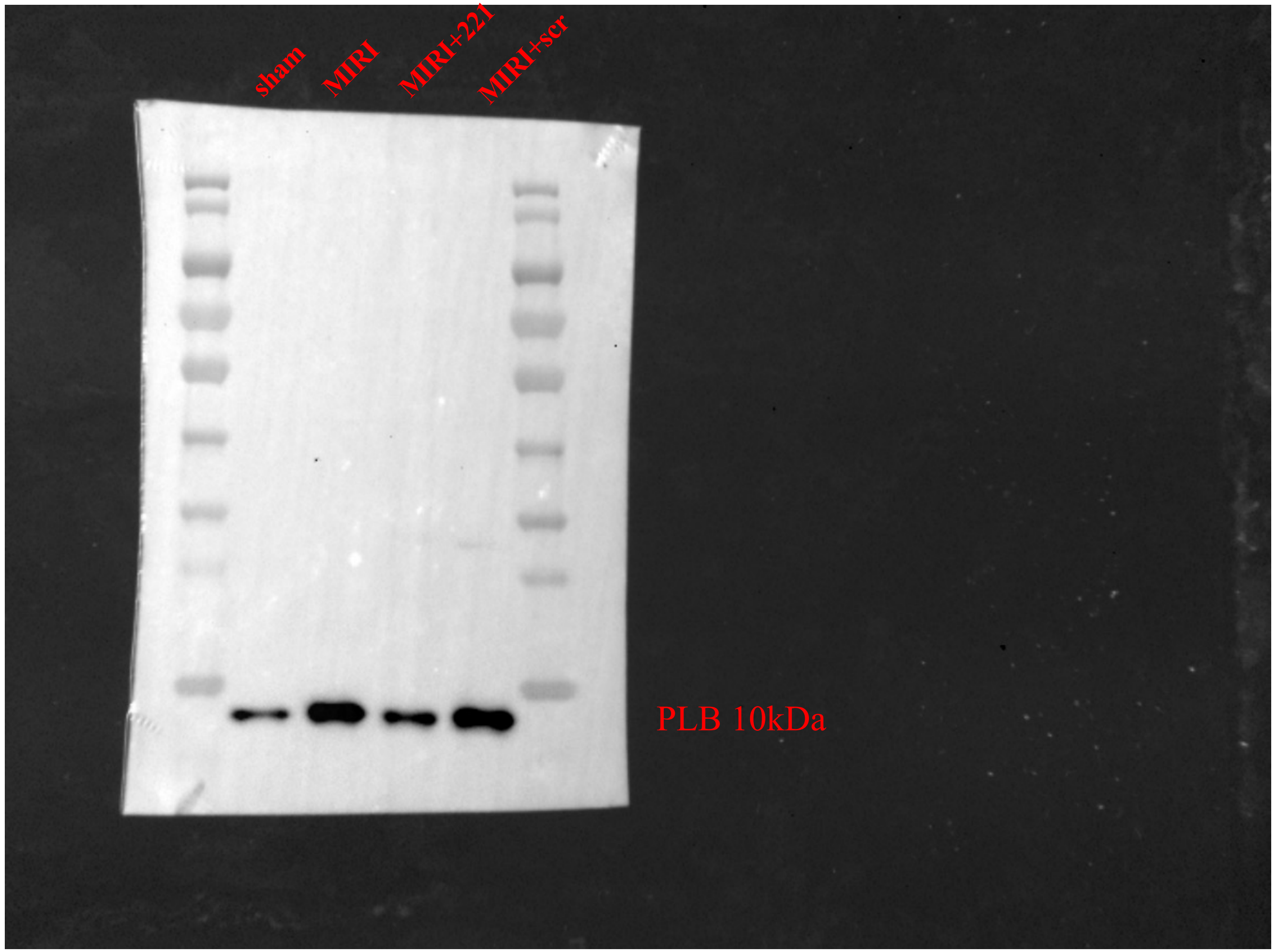

Figure 4B. PLB

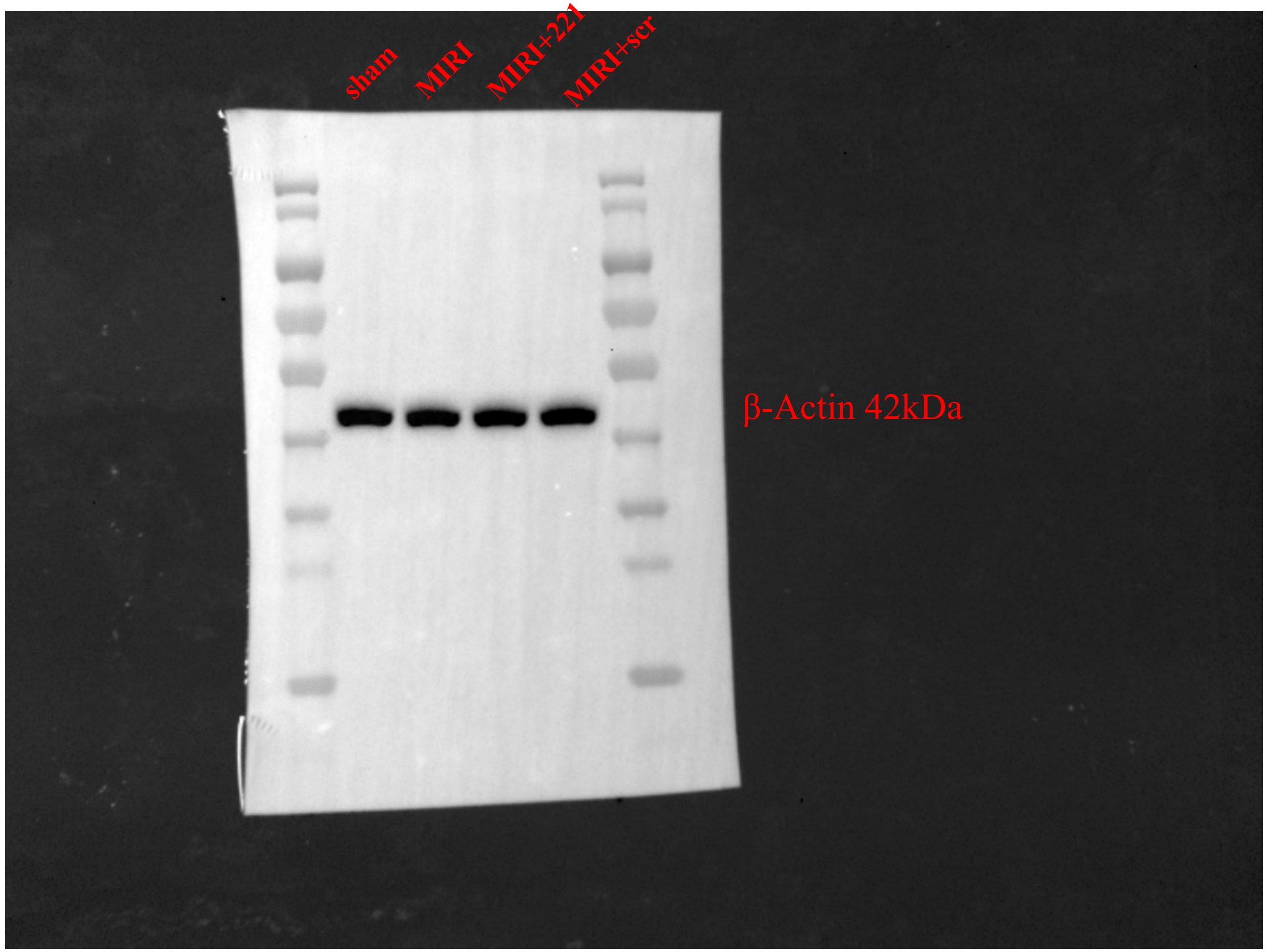

Figure 4B. β-Actin for PLB.

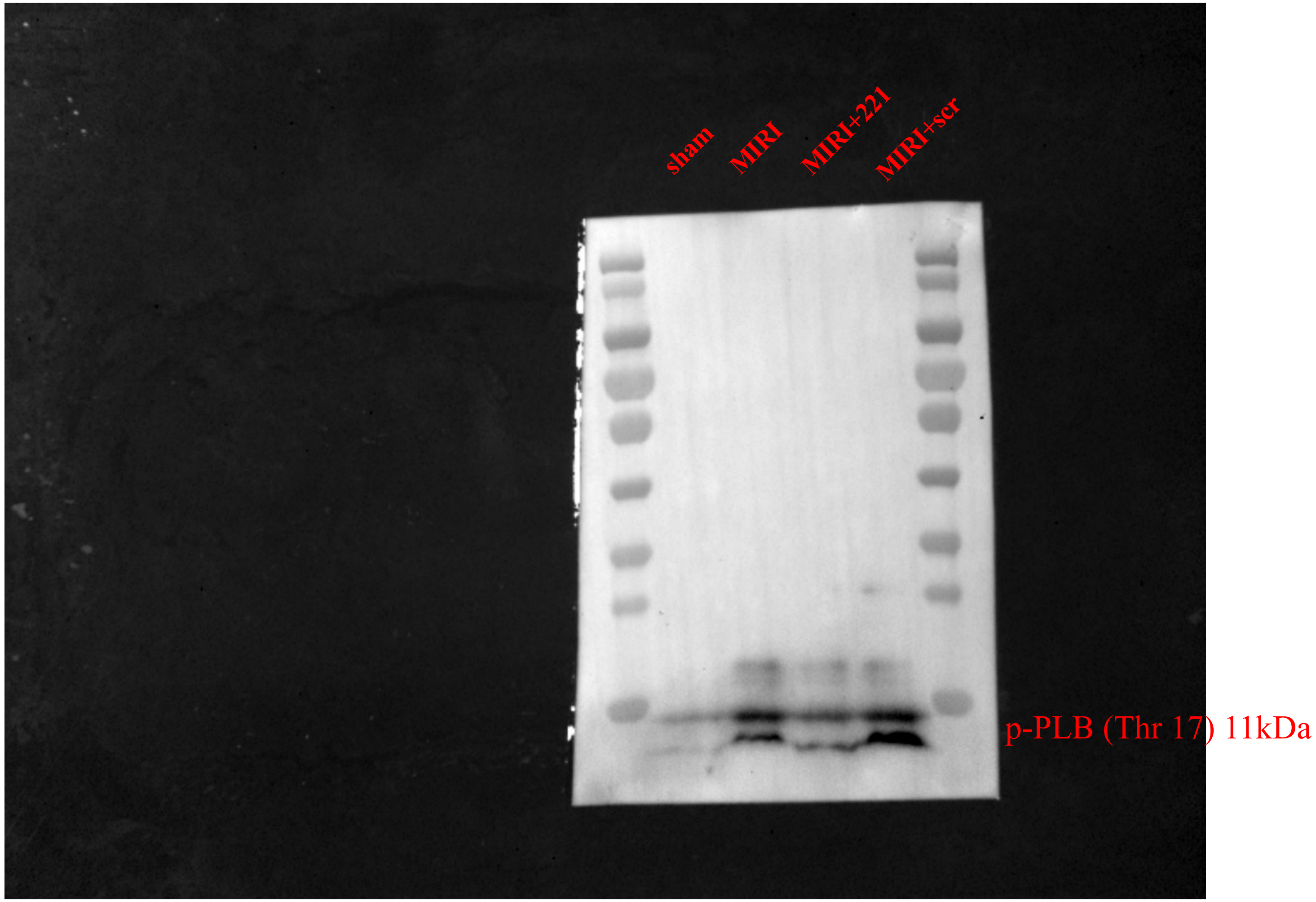

Figure 4B. P-PLB (Thr 17)

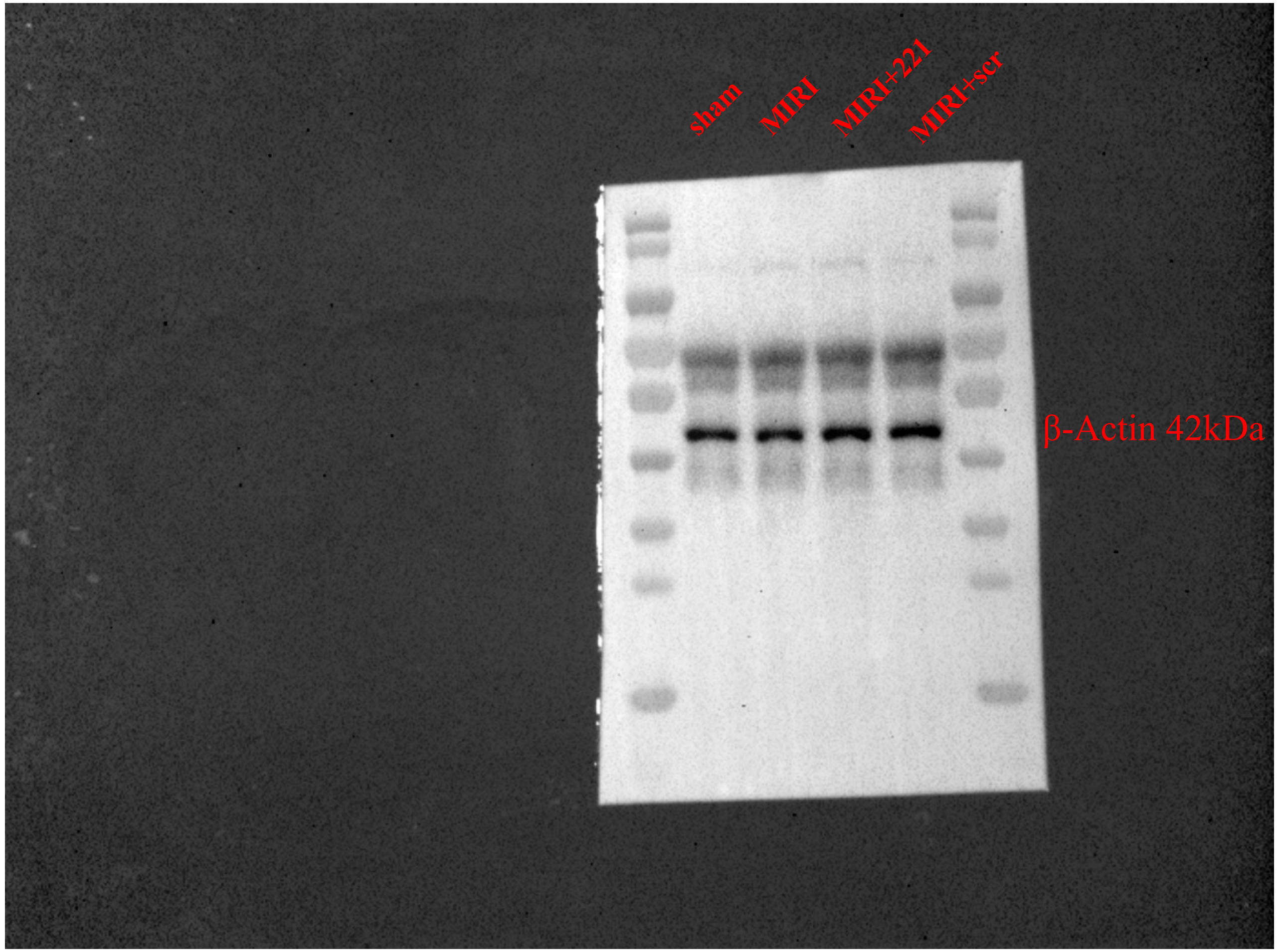

Figure 4B. β-Actin for p-PLB (Thr 17).

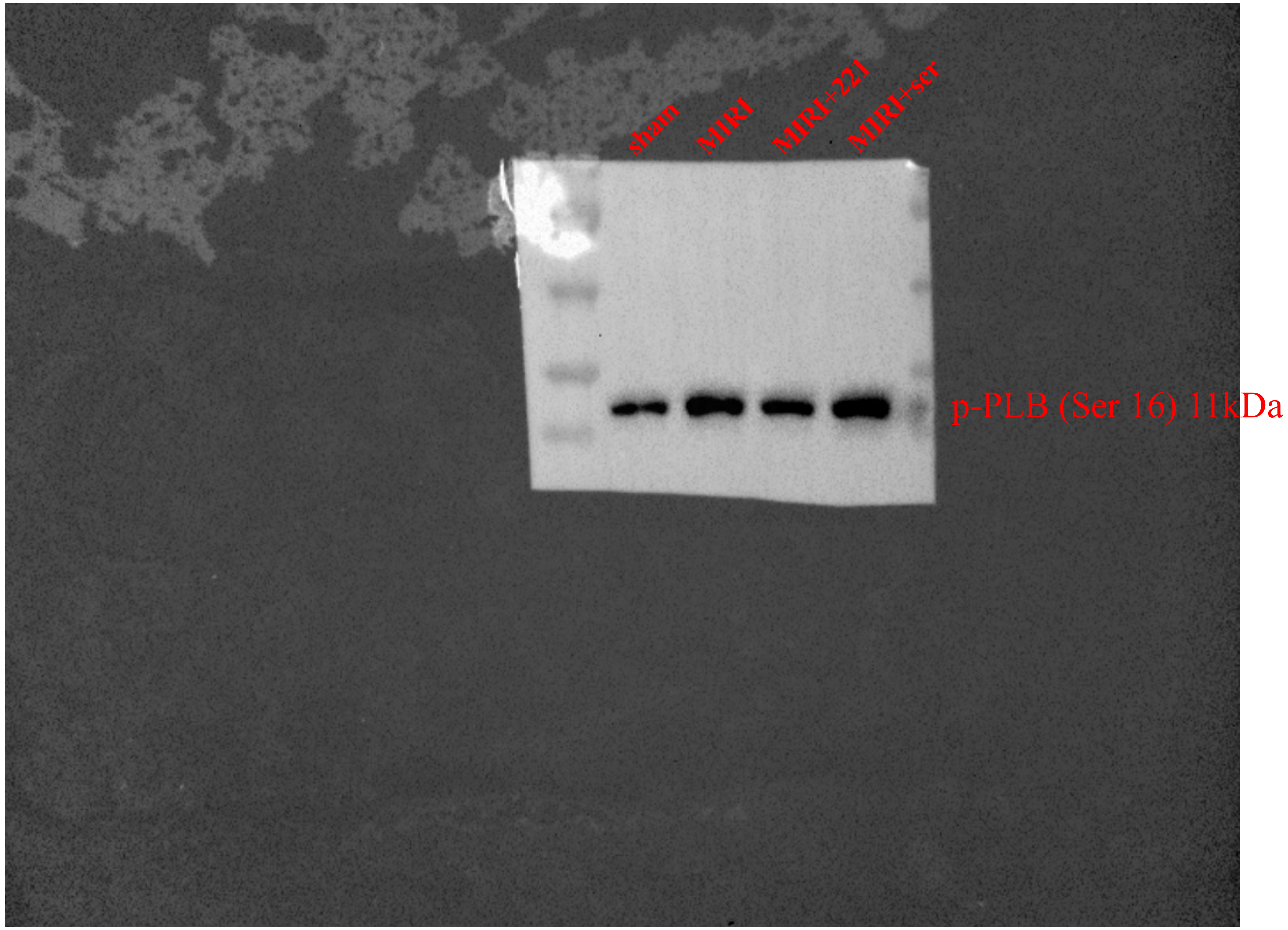

Figure 4B. p-PLB (Ser 16)

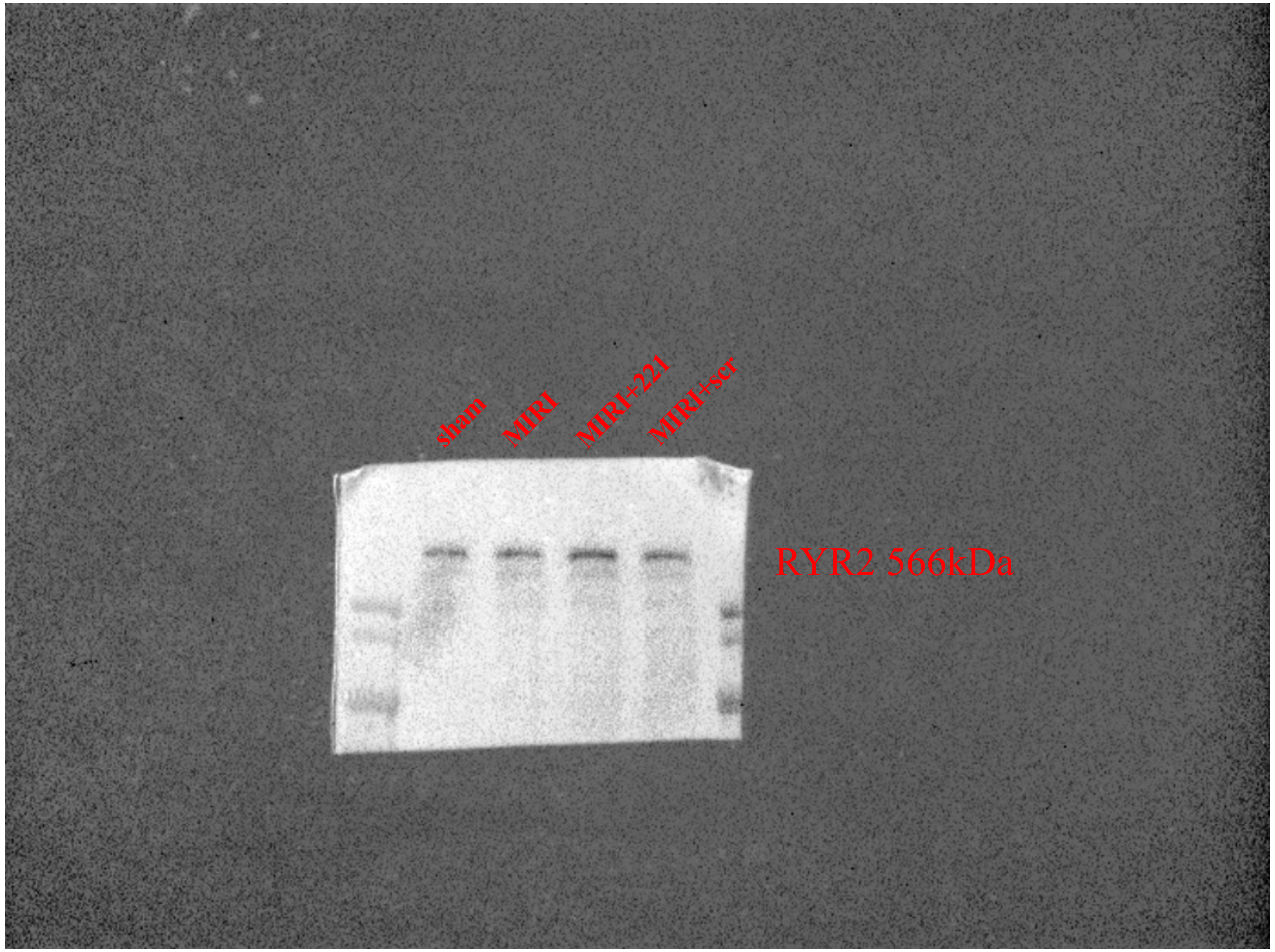

Figure 4B. RYR2

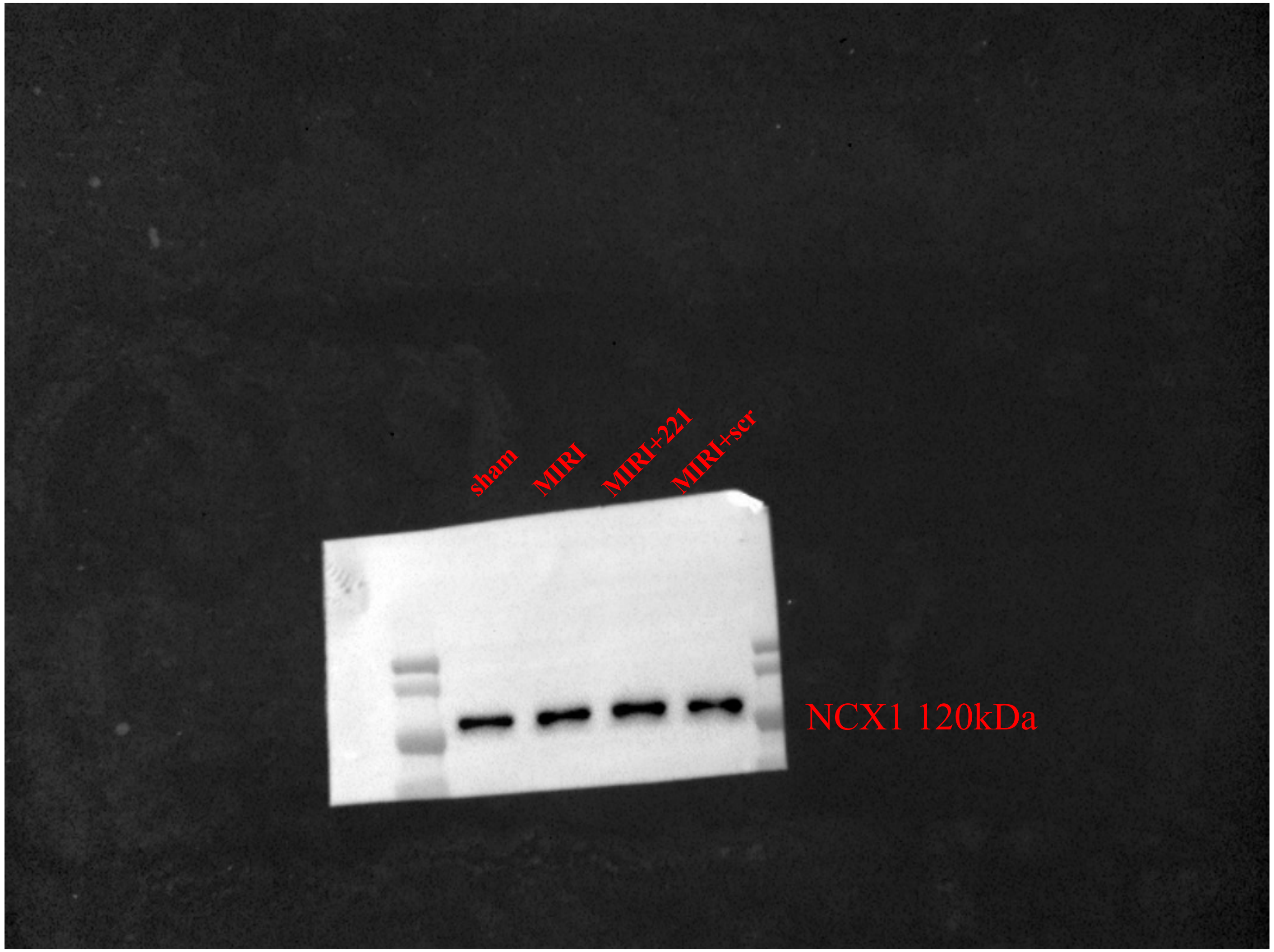

Figure 4B. NCX1

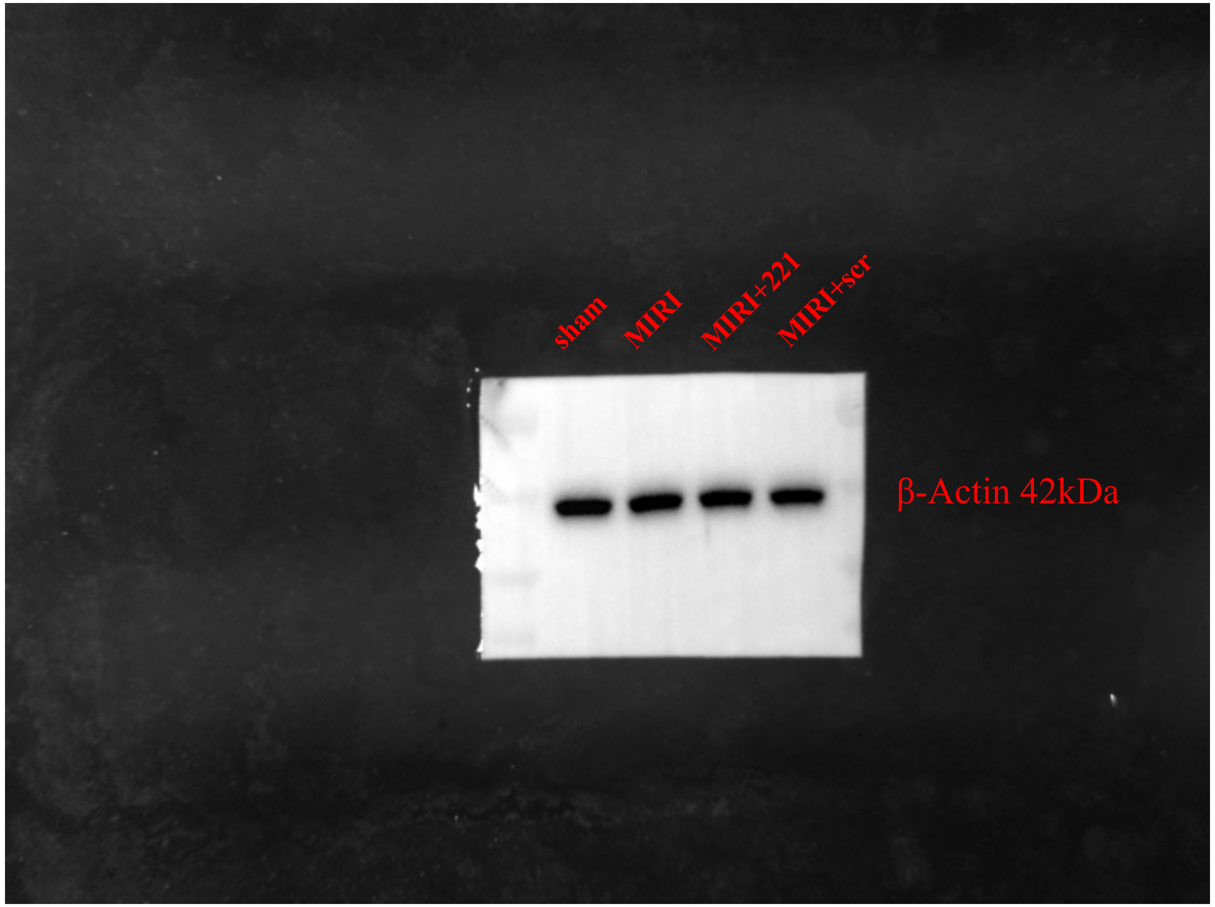

Figure 4B. β-Actin for p-PLB (Ser 16), RYR2, NCX1

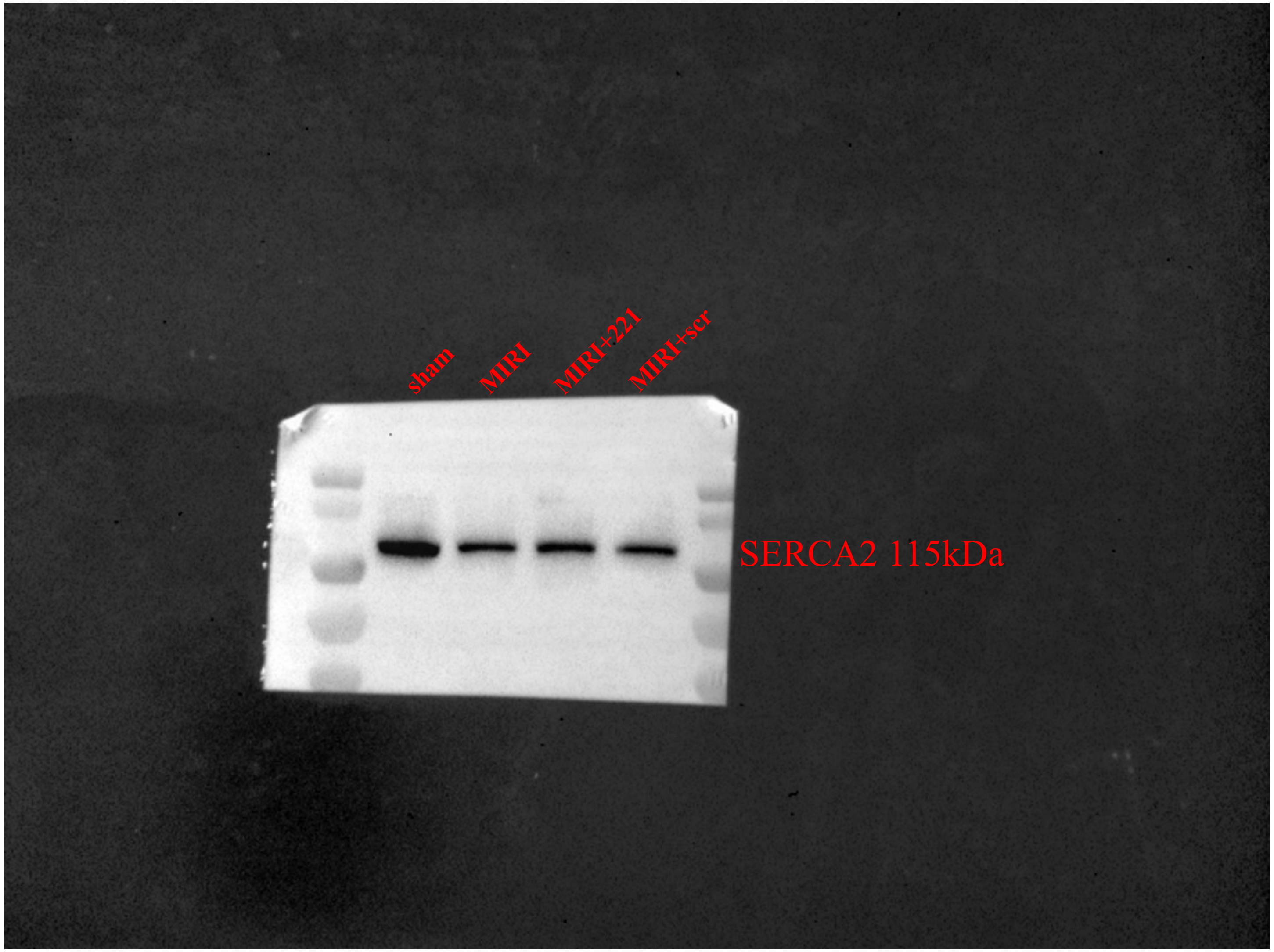

Figure 4B. SERCA2

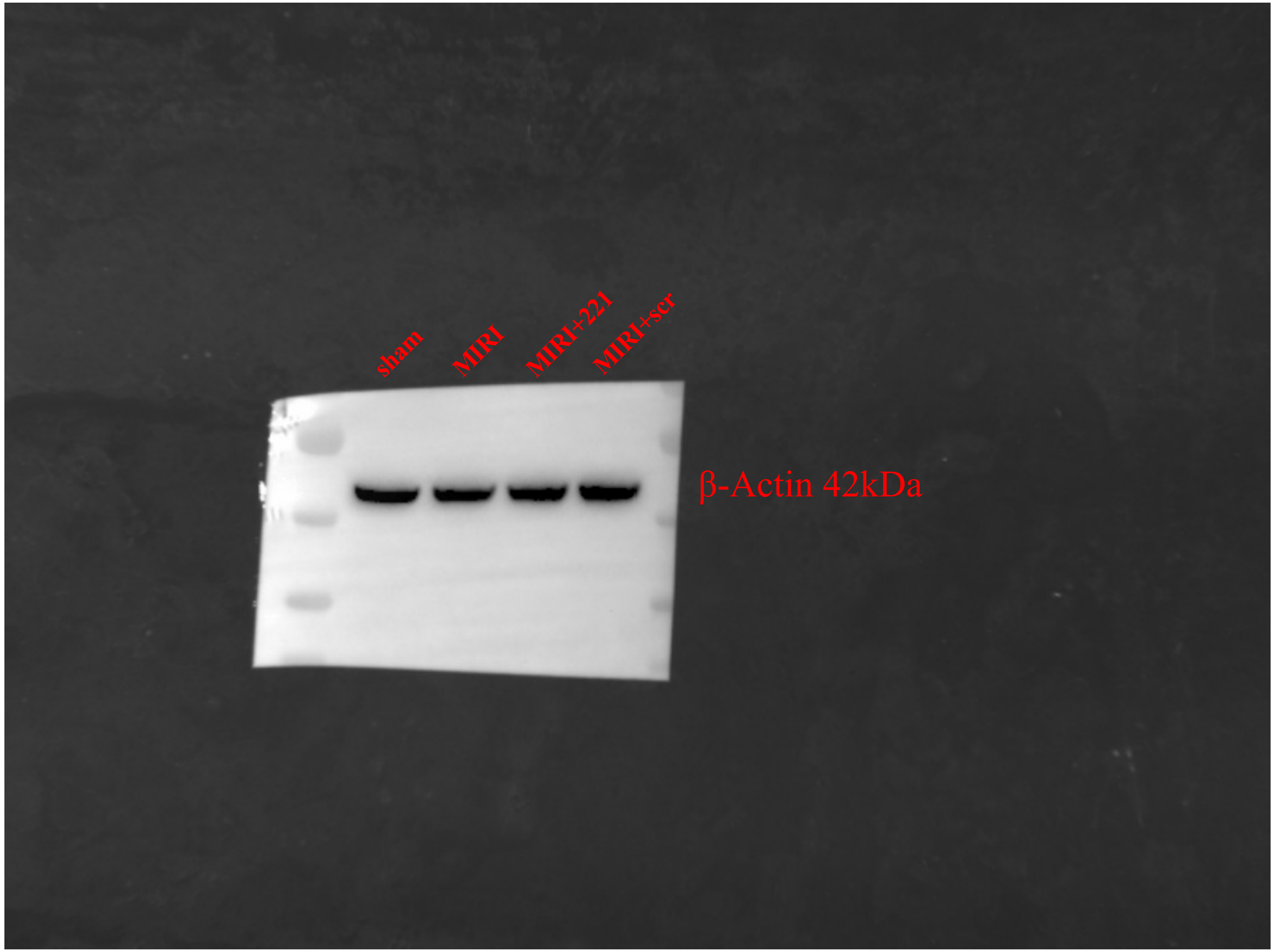

Figure 4B. β-Actin for SERCA2

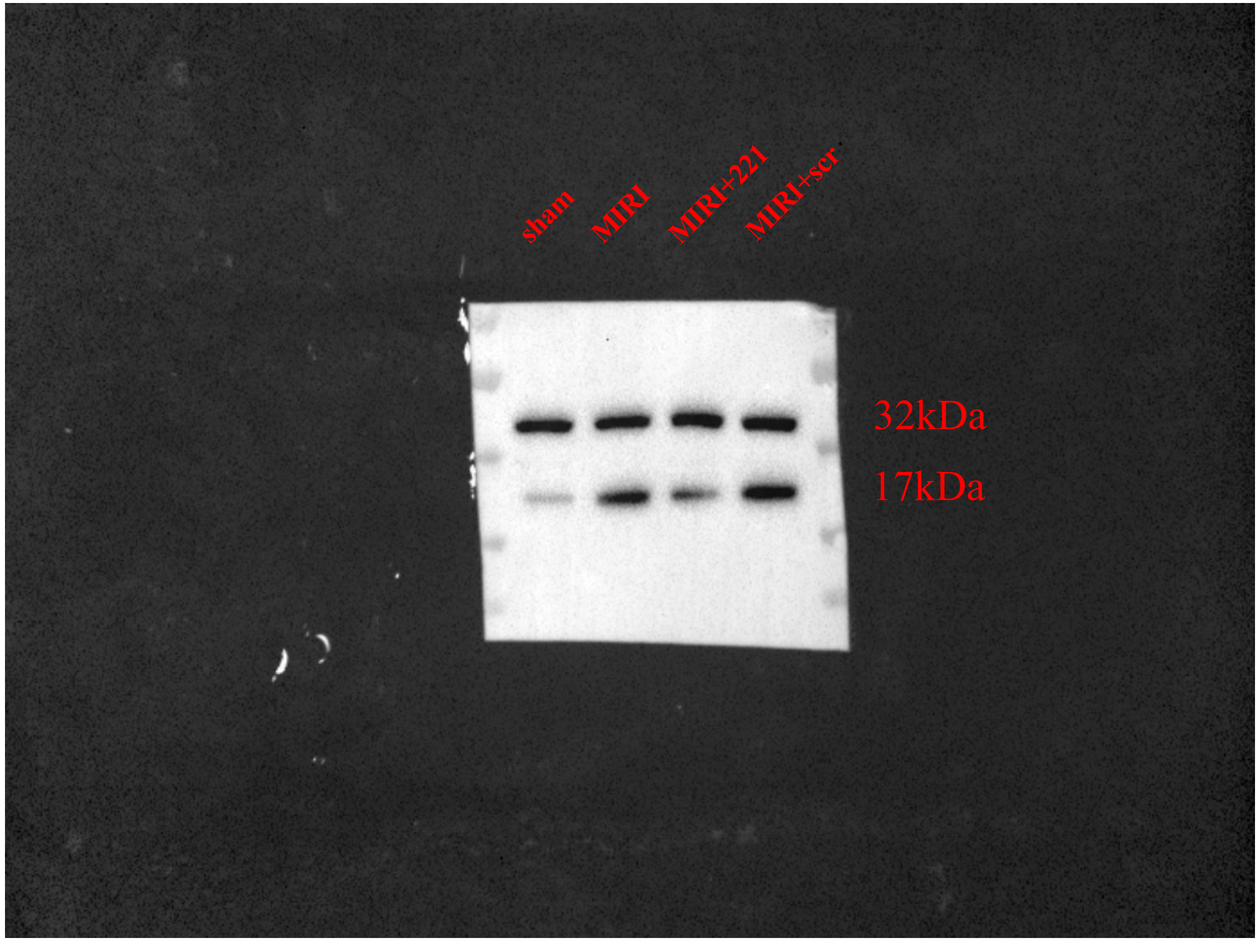

Figure 6C.caspase3

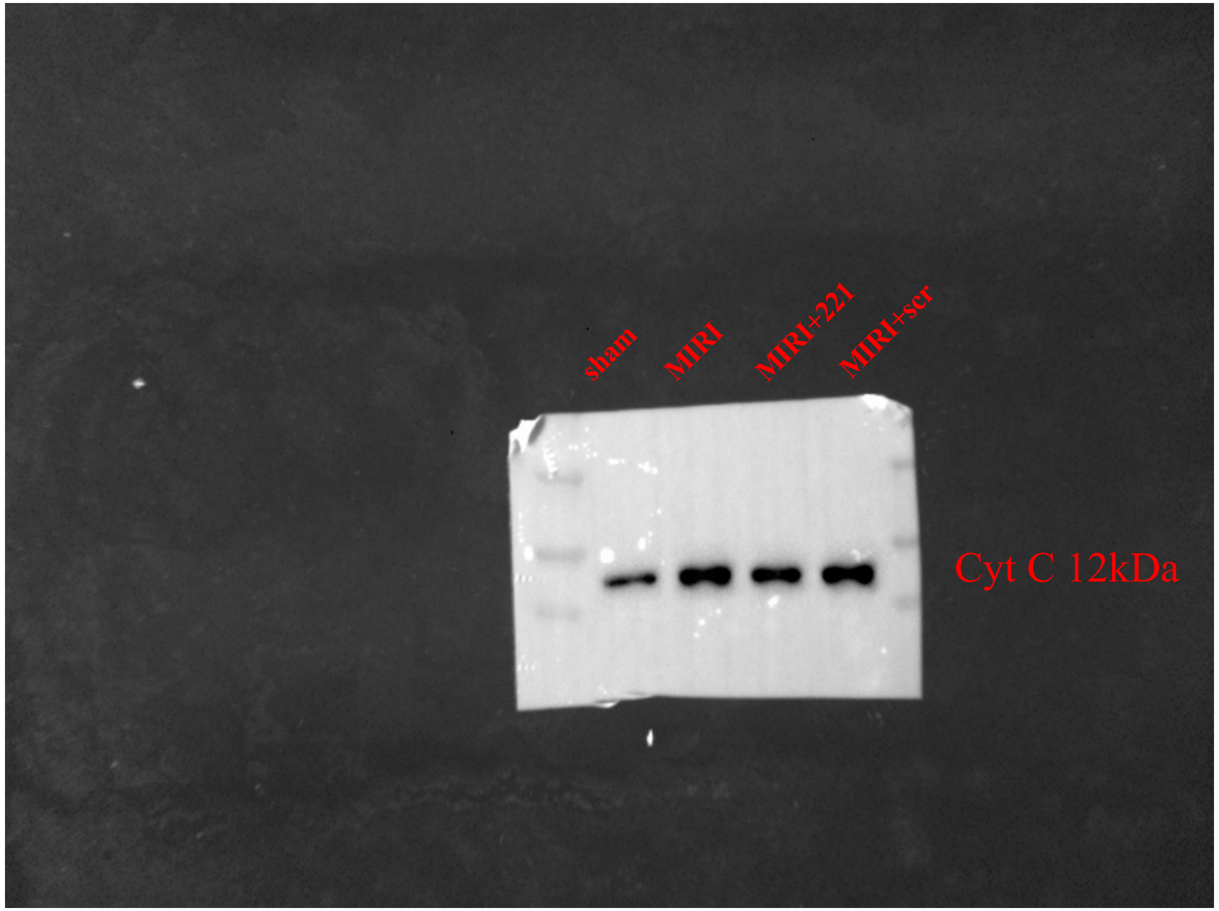

Figure 6C. Cyt C

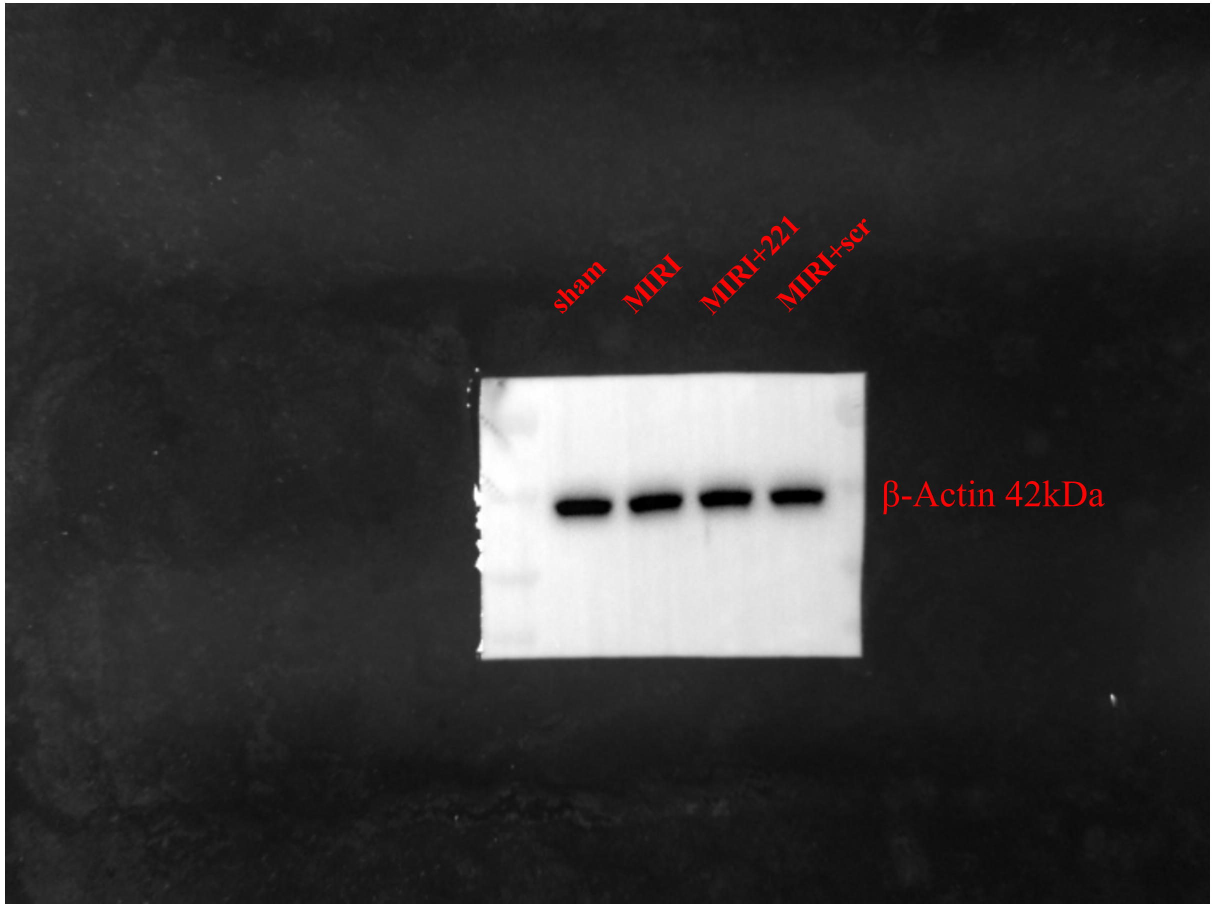

Figure 6C. β-Actin for caspase3 and Cyt C.
